# Elucidating Synergies of Single‐Atom Catalysts in a Model Thin Film Photoelectrocatalyst to Maximize Hydrogen Evolution Reaction

**DOI:** 10.1002/advs.202407598

**Published:** 2024-09-04

**Authors:** Zichu Zhao, Cheryl Suwen Law, Yanzhang Zhao, Jairo Alberto Baron Jaimez, Amin Talebian‐Kiakalaieh, Haobo Li, Jingrun Ran, Yan Jiao, Andrew D. Abell, Abel Santos

**Affiliations:** ^1^ School of Chemical Engineering The University of Adelaide Adelaide South Australia 5005 Australia; ^2^ Institute for Photonics and Advanced Sensing (IPAS) The University of Adelaide Adelaide South Australia 5005 Australia; ^3^ Department of Chemistry The University of Adelaide Adelaide South Australia 5005 Australia

**Keywords:** hydrogen evolution reactions, materials design, semiconductor thin films, single atom photoelectrocatalysts

## Abstract

Realization of the full potential of single‐atom photoelectrocatalysts in sustainable energy generation requires careful consideration of the design of the host material. Here, a comprehensive methodology for the rational design of photoelectrocatalysts using anodic titanium dioxide (TiO_2_) nanofilm as a model platform is presented. The properties of these nanofilms are precisely engineered to elucidate synergies across structural, chemical, optoelectronic, and electrochemical properties to maximize the efficiency of the hydrogen evolution reaction (HER). These findings clearly demonstrate that thicker TiO_2_ nanofilms in anatase phase with pits on the surface can accommodate single‐atom platinum catalysts in an optimal configuration to increase HER performance. It is also evident that the electrolyte temperature can further enhance HER output through thermochemical effect. A judicious design incorporating all these factors into one system gives rise to a ten‐fold HER enhancement. However, the reusability of the host photoelectrocatalyst is limited by the leaching of the Pt atom, worsening HER. Density‐functional theory calculations have provided insights into the mechanism underlying the experimental observations in terms of moderate hydrogen adsorption and enhanced gas generation. This improved understanding of the critical factors determining HER performance in a model photoelectrocatalyst paves the way for future advances in scalable and translatable photoelectrocatalyst technologies.

## Graphical abstract

Creating a rationale toward the design of high‐performing single‐atom photoelectrocatalysts. Synergistic effects combining thickness, morphology, crystallographic phase, quantity of single‐atom catalyst, and electrolyte temperature are harnessed as a design approach to maximize hydrogen evolution reaction efficiency. A mechanistic framework to describe these phenomena is also developed.

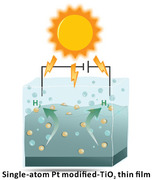

## 1 Introduction

**Figure 1:**
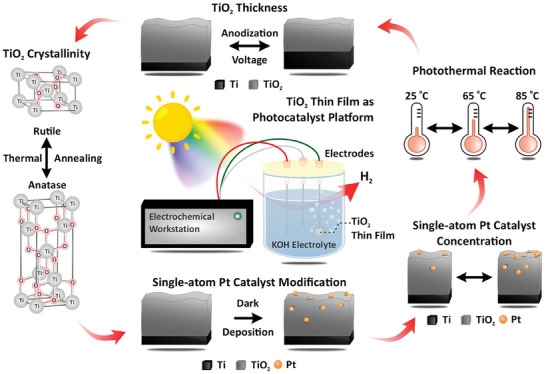
Overview of methodology to guide the design of single‐atom catalyst (SAC) systems based on nanofilms with enhanced performance for hydrogen evolution reaction (HER).

Single‐atom catalysts (SACs) based on metals have sparked extensive research across a range of emerging sustainable technologies such as electrocatalysis, photocatalysis, and photoelectrocatalysis since their discovery in 1971 and formalization in 2011.^[^ ^1^ ^]^ In combination with solid support materials, SACs act as atomically dispersed co‐catalyst inclusions to alter the lattice of the host material at a localized level.^[^ ^2^ ^]^ A combination of atomistic modification of coordination sites and defects, and spatially‐ordered distribution of isolated active sites across the host material increase the overall reactivity and selectivity of these catalytic centers for specific chemical reactions (e.g., carbon monoxide oxidation,^[^ ^3^ ^]^ carbon dioxide reduction,^[^ ^4^ ^]^ oxygen and hydrogen evolution^[^ ^5^ ^]^). The solid platform material that hosts SACs plays a critical role in determining the reactivity and selectivity of these systems by efficiently enabling charge transfer to the metal single atoms.^[^ ^6^ ^]^ As such, an ability to control and coordinate the role of the support material and SACs is crucial if we are to synthesize stable and high‐performing catalysts by regulating the surface‐free energy of isolated SACs against aggregation and clustering.^[^ ^7^ ^]^ Typical solid support platforms for SACs include carbon nitrides, covalent and metal‐ organic frameworks, carbon nanotubes, 2D materials (e.g., graphene, MXenes), and semiconductors in the form of nanoparticles.^[^ ^7^ ^]^ Although nanoparticle‐based SAC platforms provide high reactivity due to their high surface area and reduced volume, they suffer from intrinsic limitations as models to study fundamental aspects of SACs since their size distribution and composition are heterogeneous and challenging to control through conventional fabrication methods (e.g., solvothermal method).^[^ ^8^ ^]^ Alternative systems based on thin film technologies can provide better models with homogeneously controlled properties at the nanoscale. These systems can help us unravel key fundamental questions on how the local atomic configuration and dynamic changes of SACs coordinated with solid supports during reactions impact their catalytic performance and selectivity.^[^ ^9^ ^]^ Of all these platforms, solid supports based on distinct forms of TiO_2_ are considered as the benchmark material for heterogeneous photocatalysis and photoelectrocatalysis due to their high oxidative capability, highly reactive crystalline structure, chemical stability, availability, and relative cost‐competitiveness.^[^ ^10^ ^]^ To date, a broad range of TiO_2_ structures (e.g., nanoparticles, powders, nanotubes, membranes) have been widely investigated in applications such as hydrogen evolution reaction,^[^ ^11^ ^]^ bactericidal coatings,^[^ ^12^ ^]^ gas emission treatments^[^ ^13^ ^]^ and air purification.^[^ ^14^ ^]^ Of all the fabrication methods available, the generation of TiO_2_‐based structures in the form of nanotubes, nanopores, and barrier‐type (solid) nanofilms by electrochemical oxidation (anodization) of titanium has been proven to be particularly a cost‐effective, highly controllable and scalable approach.^[^ ^15^ ^]^ The high specific surface area of nanotube‐ and nanopore‐based thin films of anodic TiO_2_ modified with SACs (e.g., Pt, Pd) provide better catalytic schemes than their barrier‐type counterparts due to their high surface area and increasing reactive sites. However, these systems are not suitable to characterize the function of SACs due to their internal porous structure.^[^ ^16^ ^]^ Further, SAC modification of these structures results in a heterogeneous distribution of catalytic centers and composition associated with the restricted diffusion of SACs along their inner porous structure. In contrast, the flat and planar structure of anodic TiO_2_ nanofilms offers an ideal model system to study distinct aspects of SACs. The surface of TiO_2_ nanofilms can be homogeneously modified with SACs, and the thickness, crystallinity, and composition of the films can be precisely controlled by anodization and post‐fabrication treatments (e.g., annealing). In addition, these thin films provide a reliable substrate that can be easily characterized with benchmark techniques, making it possible to gain unique mechanistic insights into SACs (e.g., aggregation, coordination, etc.).^[^ ^17^ ^]^ TiO_2_ nanofilms can be modified with SACs through dry and wet chemistry pathways such as atomic layer deposition,^[^ ^18^ ^]^ pyrolysis,^[^ ^19^ ^]^ atom trapping,^[^ ^20^ ^]^ cyclic voltammetry,^[^ ^21^ ^]^ dark deposition,^[^ ^22^ ^]^ and photodeposition.^[^ ^23^ ^]^ Of all these methods, dark deposition is an easy‐to‐operate, effective SAC‐functionalization method for TiO_2_‐based structures,^[^ ^24^ ^]^ where SAC atoms from precursor aqueous solutions are intercalated in the oxygen vacancies of the TiO_2_ lattice. This study focuses on the development of a new model SAC system based on anodic nanofilms of TiO_2_ modified with Pt atoms via dark deposition. We use hydrogen evolution reaction (HER) as a model photoelectrocatalytic reaction to identify performance enhancements associated with structural, crystallographic, and local atomic configuration features in TiO_2_ nanofilms. The thickness, morphology, number of oxygen vacancies, and SAC distribution in these model anodic nanofilms are precisely engineered through a combination of anodization, annealing, and dark deposition. The electrochemical, chemical, crystallographic, and structural properties of these model films are comprehensively characterized to unravel the interplay of structure, SAC distribution, and level of oxygen vacancies on HER performance. We also identify synergies between the temperature of the reactant solution and the model SAC system, using HER production as an indicator. Our experimental observations are validated by a mechanistic framework using density‐functional theory (DFT) calculations. This new methodology can guide the design of SAC systems with enhanced performance for HER, using a benchmark material with precisely controlled properties at the nanoscale (**Figure** **1**).

## 2 Results and Discussion

### 2.1 Interplay of Thickness of as‐produced TiO_2_ Nanofilms and HER Performance

**Figure 2:**
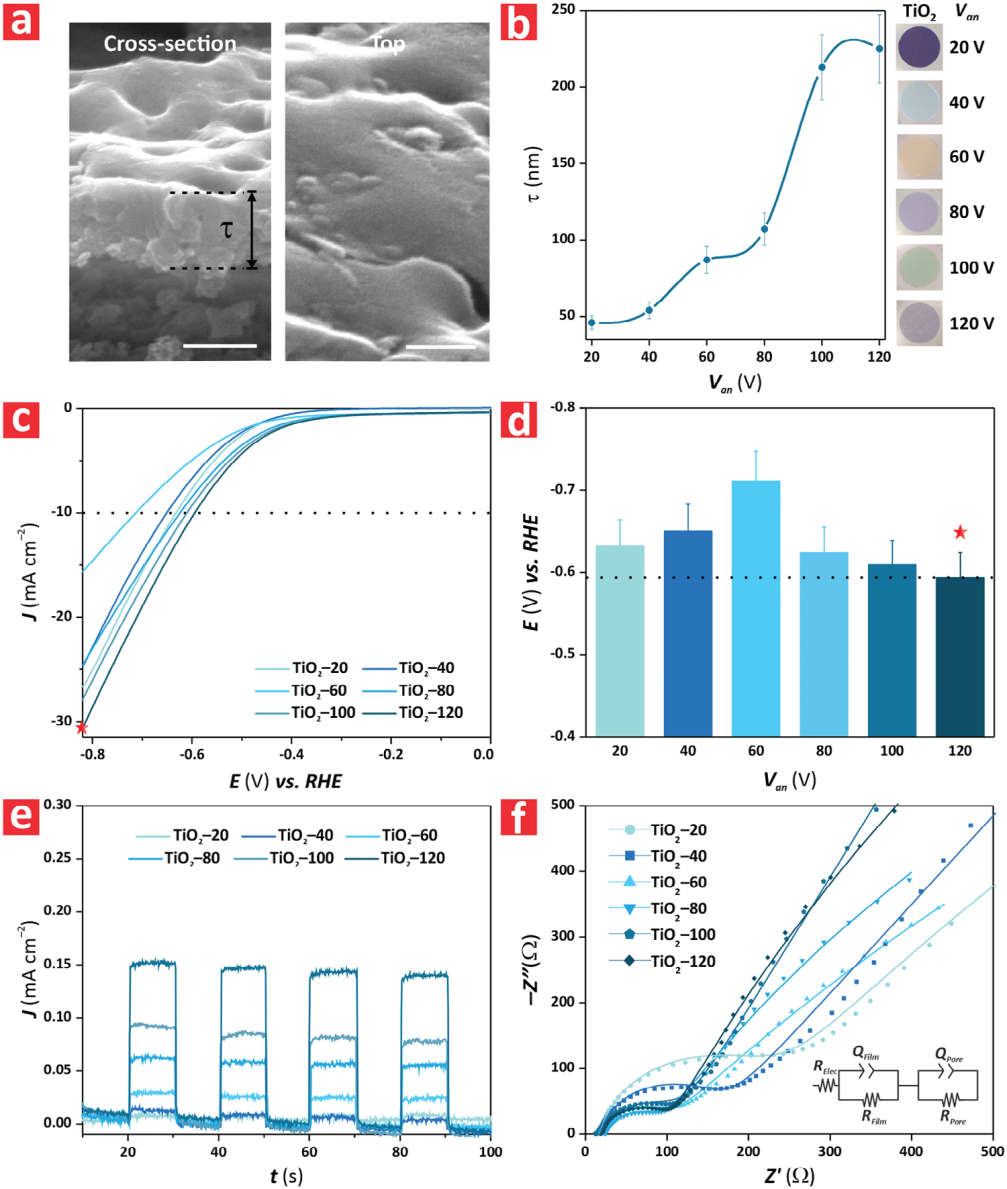
Structural and electrochemical characterization of anodic TiO_2_ nanofilms produced at varying anodization voltage (*V*_an_), from 20 to 120 V. a) Cross–sectional (left) and top (right) view FEG‐SEM images of a representative anodic TiO_2_ nanofilm fabricated at *V*_an_ = 120 V showing the characteristic morphology of these films (scale bars = 250 (left) and 100 (right) nm, respectively) (NB: data are presented as mean ± SD of a sample size of *n* ≥ 3 independent measurements). b) Correlation between anodic film thickness (*τ*) and *V*_an_ from 20 to 120 V with insets showing digital images of these films featuring vivid interferometric colors. c) Linear sweep voltammograms of anodic TiO_2_ nanofilms under illumination conditions, from TiO_2_–20 to TiO_2_–120, under varying overpotential (*E*) from −0.82 to 0.0 V versus RHE at a rate of 0.005 V s^–1^ in 1 m KOH. d) Overpotential values versus RHE measured in anodic TiO_2_ nanofilms, from TiO_2_ TiO_2_–20 to TiO_2_–120, to deliver a current density of *J* = 10 mA cm^−2^ in 1 m KOH (i.e., performance metric for HER) (NB: data are presented as mean ± SD of a sample size of *n* ≥ 3 independent measurements). e) Chronoamperometry of anodic TiO_2_ nanofilms, from TiO_2_ TiO_2_–20 to TiO_2_–120, under ON and OFF illumination modes in 1 m KOH for a period of 10 s. f) Nyquist plot of anodic TiO_2_ nanofilms in 1 m KOH, from TiO_2_ TiO_2_–20 to TiO_2_–120.

The amount of catalyst plays an important role in the catalytic performance of thin film systems.^[^ ^25^ ^]^ In anodic TiO_2_ nanofilms, the amount of catalysts over a given area is determined by the thickness of the film (*τ*), which is controlled by the anodic voltage (*V*_an_) used during the anodization process.^[^ ^26^ ^]^ Under potentiostatic conditions (i.e., *V*_an_ = constant), the anodic current density (*J_an_*) decreases exponentially with the anodization time (*t*_an_) until it asymptotically reaches a limit value close to zero, where no current flows through the system due to the electrical resistance of the film. As such, there is a maximum film thickness obtainable for a given *V*_an_, where *τ* is directly proportional to *V*_an_.^[^ ^27^ ^]^ In this study, *V*_an_ was systematically varied from 20 to 120 V with an interval (Δ*V*_an_) of 20 V for a fixed *t*_an_ of 3 min to engineer the thickness of the resultant anodic TiO_2_ nanofilms. These films were labeled as TiO_2_–20–TiO_2_–120 in reference to the anodic voltage used for their fabrication. The characteristic *V*_an_–*J_an_* versus *t*_an_ profiles of these processes are shown in Figure S1 (Supporting Information). **Figure** **2**a shows representative top and cross–sectional view FEG‐SEM images of an anodic TiO_2_ nanofilm fabricated on a Ti substrate by anodization at *V*_an_ = 120 V. Complementary top view FEG‐SEM images of TiO_2_ nanofilms can be found in Figure S2 (Supporting Information). These images revealed that TiO_2_ nanofilms had a uniform, flat surface when produced at *V*_an_ < 100 V. However, the resultant TiO_2_ nanofilms fabricated at *V*_an_ ≥ 100 V featured surface pits with an average diameter of 126 nm.^[^ ^28^ ^]^ Figure 2b depicts the relationship between *V*_an_ and the thickness of the TiO_2_ nanofilms measured via cross–sectional FEG‐SEM image analysis (Figure 2a). These measurements were further validated by optical ellipsometry. It was found that the higher *V*_an_ the thicker the anodic film was, with an estimated overall average growth rate of ≈1.875 nm V^–1^. Further analysis of this correlation revealed three distinguishable growth regimes: i) slow film growth regime from *V*_an_ = 20 to 80 V with an average linear growth rate of 0.96 ± 0.05 nm V^–1^; ii) fast film growth regime from *V*_an_ = 80 to 100 V, the average growth rate of which was 5.25 ± 0.26 nm V^–1^; and iii) a plateau growth regime from *V*_an_ = 100 to 120 V, where τ reached its maximum of 225 ± 11 nm at 120 V. Digital pictures of the resultant anodic TiO_2_ nanofilms shown in the insets of Figure 2b revealed that these films feature vivid interferometric colors, which are directly correlated with the film thickness. The photoelectrocatalytic performance of anodic TiO_2_ nanofilms for HER as a model reaction was characterized in a three‐electrode system using 1 m KOH aqueous electrolyte, where the anodic TiO_2_ nanofilm was the working electrode. Figure 2c shows the evaluation of the photoelectrocatalytic HER of TiO_2_–20–TiO_2_–120 nanofilms at room temperature in 1 m KOH performed by linear sweep voltammetry (LSV) under illumination conditions. The current density (*J*) output of these films upon application of an external bias voltage, from −0.82 to 0.00 V versus RHE, was characterized by an exponential decay until *J* reached a value of ≈0 mA cm^−2^ at *V* = 0 V versus RHE. To elucidate the best‐performing nanofilm in terms of HER, the overpotentials (*E*) of these working electrodes versus RHE to deliver a current density of *J* = 10 mA cm^−2^ (i.e., *η*_10_ as a performance metric for HER) are shown in Figure 2d. It was apparent from these results that *E* increased from TiO_2_–20 (−0.63 ± 0.03 V) to TiO_2_–60 (−0.71 ± 0.04 V), and then decreased until reaching a minimum value of −0.59 ± 0.03 V for the TiO_2_–120 nanofilm. It is worth noting that lower overpotential means that less input energy is required to generate electron–hole pairs at a fixed current density, which is therefore a desired feature for photoelectrocatalytic applications. The trend seen in Figure 2d can be attributed to a combined effect of the thickness and morphology of TiO_2_ nanofilms. When the thickness of the nanofilm was increased by *V*_an_ within the range of 20–60 V, the transfer of electrons from the bulk of the film to the surface was slowed down due to efficient electron–hole recombination. However, after the model anodic film reached a critical thickness (i.e., TiO_2_–60, *τ* = 213 ± 7 nm), pits generated on the top surface of the anodic film increased the effective surface area available for HER reaction. This in turn led to an enhancement in the activity of HER reaction, where the best performance was achieved by the TiO_2_–120 nanofilm. Figure S3a–c (Supporting Information) shows the cyclic voltammetry at different scan rates (i.e., 20, 40, 60, 80 and 100 mV s^–1^) of model TiO_2_ nanofilms produced at anodic voltages from 80 to 120 V. These were selected because of the gradual formation of pits on the surface of the nanofilms from 80 V. Figure S3d (Supporting Information) compiles the summary of the electrochemical surface area (ECSA) values estimated from these graphs, which revealed an increment of ECSA with *V_An_* from 13.98 ± 1.40 to 22.06 ± 2.21 cm^2^ cm^−2^ for TiO_2_–80 and TiO_2_–120 nanofilms, respectively. This result is in good agreement with our FEG‐SEM analysis, where there is an apparent increase in the formation of pits on the surface of these semiconductor nanofilms with the anodizing potential from 80 V. The UV–Vis diffuse reflectance spectrum of a model TiO_2_–120 nanofilms (Figure S4a, Supporting Information) revealed that these nanofilms have an energy band gap of ≈3.93 eV, as estimated from the Schottky diagram (Figure S4b, Supporting Information). This observation was further confirmed by electrochemical impedance spectroscopy measurements (vide infra). Figures S5 and S6 (Supporting Information) show the electrocatalytic performance of anodic TiO_2_ nanofilms under non‐illuminated conditions, and a comparison of overpotentials at *J* = 10 mA cm^−2^ under illumination (photoelectrocatalysis, PEC) and dark conditions (electrocatalysis, EC), respectively. These results revealed that, under both PEC and EC configurations, the HER performance of the anodic films followed comparable trends, where the best‐performing nanofilm was TiO_2_–120. These results further confirmed the existence of a critical film thickness value (i.e., TiO_2_–60) beyond which the HER performance improved with *τ*. As expected, the PEC systems outperformed their EC counterparts due to the additional electron–hole pairs generated by the semiconductor films under illumination conditions (i.e., less input energy was required to generate the same amount of electron–hole pairs for a given current density value) and the efficient separation of charge carriers. Further analysis of these graphs indicated that the difference of overpotentials between illuminated (ON) and non‐illuminated (OFF) conditions (i.e., Δ*E* = *E*_ON_−*E*_OFF_) increased with *τ*, with values of Δ*E* = 0.045 ± 0.005 V and 0.034 ± 0.003 V from TiO_2_–20 to TiO_2_–120 nanofilms, respectively (Figure S7, Supporting Information).

The increment in Δ*E* between PEC and EC settings suggested that the active sites in the anodic TiO_2_ films increased with the anodizing potential, leading to an enhancement in HER activity that can be attributable to the presence of pits on the surface of the films. Figure 2e illustrates the photocurrent generated by the model TiO_2_ nanofilms upon successive ON–OFF illumination cycles. It is apparent from these graphs that these films responded instantaneously to the input light stimulus, generating a photoanodic current that was dramatically higher than that recorded under dark conditions (i.e., *J* ≈0 mA cm^−2^). Further analysis of the difference in photocurrent between ON and OFF conditions (i.e., Δ*J* = *J_ON_*–*J_OFF_*) indicated that TiO_2_–20 and TiO_2_–40 films showed the lowest response to light, with comparable values of Δ*J* (i.e., 0.0055 ± 0.0006 and 0.0090 ± 0.0009 mA cm^−2^, respectively). However, there was an apparent exponential increment of Δ*J* with *τ*, from TiO_2_–60 to TiO_2_–120, where the thicker anodic film achieved the highest level of response to light with a value of Δ*J* = 0.1410 ± 0.0141 mA cm^−2^ (Figure S8, Supporting Information). Figure 2f shows the electrochemical impedance spectroscopy (EIS) analysis of the model anodic TiO_2_ nanofilms. It was found that the film TiO_2_–60 attained the highest charge transfer resistance across the electrode–electrolyte interface, which was in good agreement with the poor HER performance achieved by these nanofilms (Figure 2c,d). Another observation was that the impedance of the films from TiO_2_–80 to TiO_2_–120 were similar up to 100 Ω. Beyond this point, the gap in the impedance of these films increased. This result may have been caused by the effect of mass transfer and film architecture. Further insights into EIS measurements obtained from equivalent circuit fittings (Table S1, Supporting Information) revealed that anodic TiO_2_ nanofilms could be mechanistically described by a system that considered the electrolyte resistance, the film resistance and capacitance, the charge transfer resistance, and a double‐layer capacitance. These results further confirmed the values of overpotential estimated for these model nanofilms at *η*_10_. **Table** **1** shows a comparison with other forms of as‐produced TiO_2_ structures. This analysis reveals that the performance of anodic TiO_2_ nanofilms in terms of photocurrent density is comparable to that of other existing systems.^[^ ^29^, ^30^, ^31^, ^32^ ^]^

**Table 1:** Performance comparison of recently reported TiO_2_‐based HER photocatalysts in terms of photocurrent density.

Composite	Photocurrent density	Electrolyte	References
TiO_2_ thin films	0.56 mA cm^−2^	1 m NaOH (1.23 V vs RHE)	[29]
TiO_2_ Nanotubes	1.6 mA cm^−2^	0.1 m KNO_3_ (0.6 V vs Ag/AgCl)	[30]
NiO/TiO_2_	1.68 mA cm^−2^	0.1 m KOH (1.23 V vs RHE)	[31]
Au modified TiO_2_	3.7 mA cm^−2^	1 m KOH (0.187 V vs RHE)	[32]

### 2.2 Impact of Annealing Temperature of TiO_2_ Nanofilms on HER Performance

**Figure 3:**
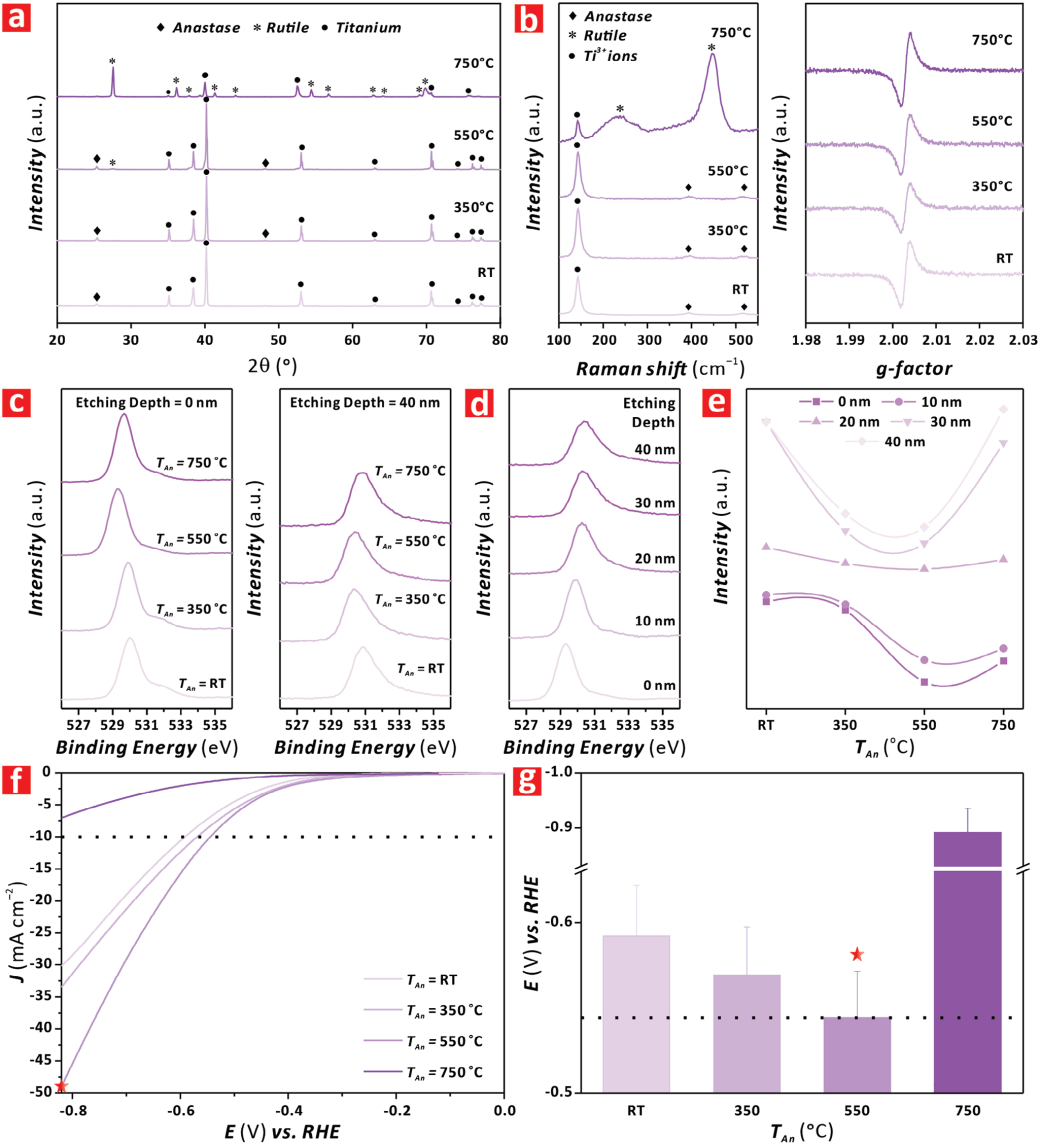
Crystallographic and electrochemical characterization of anodic TiO_2_ nanofilms subjected to distinct annealing temperatures (*T*_An_ = RT, 350, 550, and 750 °C). a) XRD spectra of TiO_2_–120 nanofilms annealed at *T*_An_ = RT, 350, 550, and 750 °C with legends indicating the characteristic peaks of anatase, rutile, and titanium. b) Raman (left) and EPR (right) spectra of TiO_2_–120 nanofilms annealed at *T*_An_ = RT, 350, 550 and 750 °C. c) O 1s XPS spectra of TiO_2_–120 nanofilms annealed at *T*_An_ = RT, 350, 550, and 750 °C performed on the surface of the nanofilm (i.e., etching depth = 0 nm). d) O 1s XPS spectra of TiO_2_–120 nanofilms annealed at *T*_An_ = RT, 350, 550, and 750 °C performed on the bulk of the nanofilm (i.e., etching depth = 40 nm). e) O 1s XPS relative intensity area at 531.6 eV (i.e., oxygen vacancies band) of TiO_2_–120 nanofilms annealed at *T*_An_ = RT, 350, 550, and 750 °C performed at distinct depths along the nanofilm thickness, from 0 to 40 nm at steps of 10 nm. f) Linear sweep voltammograms of anodic TiO_2_–120 nanofilms annealed at *T*_An_ = RT, 350, 550, and 750 °C under varying overpotential (*E*) from −0.82 to 0.0 V versus RHE at a rate of 0.005 V s^–1^ in 1 m KOH. g) Overpotential values versus RHE measured in anodic TiO_2_–120 nanofilms at *η*_10_ in 1 m KOH (NB: data are presented as mean ± SD of a sample size of *n* ≥ 3 independent measurements).

The photoelectrocatalytic HER performance of TiO_2_‐based structures is highly determined by the crystallographic phase of the semiconductor.^[^ ^33^ ^]^ The crystalline structure of as‐produced anodic TiO_2_ nanofilms is amorphous. However, post‐fabrication annealing makes it possible to finely tune the crystalline structure of these anodic TiO_2_ nanofilms to increase the number of active catalytic sites and boost HER.^[^ ^34^ ^]^ To gain insights into the effect of crystallization on the HER performance of anodic TiO_2_ nanofilms, a model TiO_2_–120 nanofilms—best‐performing structure for HER—was subjected to a range of annealing temperatures (i.e., *T*_An_ = room temperature RT, 350, 550 and 750 °C). X‐ray diffraction (XRD) spectra shown in **Figure** **3**a indicated that the structure of as‐produced TiO_2_ nanofilms was composed primarily of titanium dioxide with an almost negligible characteristic diffraction peak of anatase phase at 2*θ*= 25.37°. The residual amount of anatase in these TiO_2_ nanofilms can be attributed to microcrystals formed by localized Joule heat during anodization at high voltage. This was confirmed by the Raman spectra of TiO_2_ nanofilms produced at *V*_an_ = 20–120 V (Figure S9, Supporting Information). When the nanofilms were annealed at 350 °C, they consisted of an anatase phase with its characteristic diffraction peaks at 2*θ* = 25.37 and 48.1°. Upon annealing at 550 °C for 1 h, the TiO_2_ in the anodic nanofilms turned into a mixture of anatase and rutile phases, where the latter phase had its characteristic peak at 2*θ* = 27.5°. When *T*_An_ reached 750 °C, the TiO_2_ nanofilms were entirely converted into a rutile phase, where a dramatic increase in intensity was found at 2*θ* = 27.5° when compared to the same peak in the nanofilm annealed at *T*_An_ = 550 °C. Figure 3b (left) shows the Raman spectra of TiO_2_ nanofilms at distinct annealing temperatures, from *T*_An_ = RT to 750 °C. These results further corroborated our observations by XRD. The Raman shifts at 144, 396 and 516 cm^−1^ can be assigned to anatase phase, whereas those Raman shift bands located at 250 and 447 cm^−1^ were attributable to rutile phase.^[^ ^35^ ^]^ It was found that the band associated with O–Ti–O bonds at 144 cm^−1^ slightly increased when *T*_An_ was increased from RT to 550 °C. This result was associated with a reorganization of the O–Ti–O bonds from amorphous TiO_2_ to its anatase phase. Upon further increment of *T*_An_ from 550 to 750 °C, there was a dramatic decrease in intensity of the Raman shift band at 144 cm^−1^. Conversely, the characteristic bands of rutile phase appeared in the Raman spectrum at 250 and 447 cm^−1^. Figure S10 (Supporting Information) shows top‐view FEG‐SEM images of these films treated at distinct *T*_An_, which confirmed that the changes in crystal structure, from amorphous to rutile when *T*_An_ was increased from 350 to 750 °C, were accompanied by morphological changes in the nanofilms. Whereas anodic TiO_2_ nanofilms annealed at *T*_An_ = RT and 350 °C kept their characteristic flat surface morphology with pits, their analogs treated at *T*_An_ = 550 and 750 °C underwent significant morphological changes. The nucleation of small clusters of rutile crystals across the top surface of the anodic film was apparent at *T*_An_ = 550 °C. However, it can be observed that there was a significant change in the size and dimensions of rutile crystals at *T*_An_ = 750 °C. This extreme crystal growth resulted in the complete destruction of the surface pits in the films. According to Schmuki et al,^[^ ^36^ ^]^ thermal annealing of anodic TiO_2_ may contain ample intrinsic structural defects. To further analyze the structure of annealed TiO_2_ nanofilms, electron paramagnetic resonance (EPR) at an X‐band frequency of 9.8 GHz was used to detect unpaired electrons in paramagnetic species. This is a powerful method to identify the existence of Ti^3+^, oxygen vacancies, and other structural defects, and how these can affect the HER activity of these model structures. Figure 3b (right) shows the EPR spectra measured at room temperature without the illumination of the model TiO_2_–120 nanofilms treated at *T*_An_ = RT, 350, 550, and 750 °C. These results indicated that all TiO_2_ nanofilms exhibited comparable EPR resonances, which were characterized by low‐intensity signals arising from lattice‐embedded Ti^3+^ sites with no paramagnetic species existing in the structure of TiO_2_.^[^ ^37^ ^]^ The apparent symmetrical signals at a value of g‐factor = 2.003 in the EPR spectra of TiO_2_ nanofilms at RT, 350, 550 to 750 °C were attributable to oxygen vacancies (with one electron). The intensity of the EPR spectral signal increased with *T*_An_ and the concurrent generation of oxygen vacancies in the structure of TiO_2_ due to the crystalline transition, from amorphous to anatase and from anatase to rutile phases. The intensity of the g‐factor at 2.003 for these model TiO_2_ nanofilms increased from RT to 750 °C, reaching its maximum at 750 °C. Figure 3c shows the O 1s X‐ray photoelectron spectroscopy (XPS) spectra of TiO_2_ nanofilms annealed at distinct temperatures (i.e., RT, 350, 550 to 750 °C) and depth along the nanofilm thickness (i.e., from 0 nm (surface) to 40 nm, at steps of 10 nm). The amount of oxygen vacancies were quantified by analyzing the relative area of the oxygen vacancy band at 531.6 eV.^[^ ^38^ ^]^ This analysis indicated that the amount of oxygen vacancies decreased with increasing temperature, from RT to 550 °C, and slightly increased from 550 to 750 °C. This result was aligned with the EPR spectra shown in Figure 3b. Figure 3d shows the O 1s XPS spectra of TiO_2_ nanofilms at RT, 350, 550 to 750 °C obtained at varying depths along the nanofilm thickness through a combination of ion milling and XPS analysis, from 0 to 40 nm. It can be observed that the number of oxygen vacancies increased along the nanofilm thickness at any annealing temperature. This in‐depth variation in crystallographic structure in the form of oxygen vacancies along the nanofilm thickness can be attributed to the existence of a thermal gradient between the surface and the bulk of the nanofilms when these were exposed to the thermal treatment. The atoms located at the surface of the film re‐arrange their structure at a faster rate than those located within the bulk of the nanofilm. Figure 3e depicts a summary of the relative area of oxygen vacancies band in TiO_2_ nanofilms analyzed at RT, 350, 550 to 750 °C, from 0 to 40 nm etching depth, whereas Figure S11 (Supporting Information) shows the raw O 1s XPS spectra. Evaluation of HER reaction for the model TiO_2_–120 nanofilms at *T*_An_ = RT, 350, 550, and 750 °C showed that the best performance was achieved by the TiO_2_–120 nanofilms annealed at 550 °C (Figure 3f). The current density at the lowest overpotential (i.e., *E* = −0.82 V) was found to be −30.0, −33.5, −48.9, and −7.1 mA cm^−2^ for TiO_2_–120 nanofilms annealed at RT, 350, 550 and 750 °C, respectively. This result may be attributed to the contribution of anatase phase crystalline structure, which contains higher surface adsorption capacity for hydroxyl groups and provides one order of magnitude longer lifetime of photogenerated electron–hole pairs than that of rutile structures.^[^ ^39^ ^]^ It has also been reported that the rutile phase has a detrimental effect on the electron diffusion length in TiO_2_ nanotubes.^[^ ^40^ ^]^ The overpotential of the TiO_2_–120 nanofilm annealed at 550 °C was measured to be *E* −0.54 ± 0.03 V at *η*_10_, whereas its counterparts annealed at *T*_An_ = RT, 350 and 750 °C reached values of −0.59 ± 0.03, −0.60 ± 0.03, and −0.89 ± 0.04 V (Figure 3g). This result further indicated that the TiO_2_ nanofilms annealed at 550 °C attained the best performance within the range of annealing temperature assessed. This performance enhancement can be explained in terms of excited electrons trapped in oxygen vacancies, where these are easier to transfer to external atoms for PEC in anatase TiO_2_ nanofilms than in the case of rutile phase structures due to the deep trapped states.^[^ ^41^ ^]^ This result was also well‐aligned with the results reported in Figure 3b,c regarding the number of oxygen vacancies quantified in these nanofilms.

Figures S12 and S13 (Supporting Information) show the electrocatalytic performance of annealed TiO_2_ nanofilms under non‐illuminated conditions, and a comparison of overpotentials at *η*_10_ under ON and OFF illumination states, respectively. It was found that there is a decreasing trend in *E* with *T_An_* for the ON‐ and OFF‐illumination states, where the minimum required overpotential at *η*_10_ was achieved by the TiO_2_–120 nanofilms annealed at 550 °C. It was also apparent that the Δ*E* = *E_ON_*–*E_OFF_* was relatively constant within the *T_An_* range assessed in this study, with an average value of 0.047 ± 0.005 V. Altogether, these analyses indicated that the optimal annealing temperature for TiO_2_–120 nanofilms in terms of HER performance was 550 °C.

### 2.3 Engineering of Pt SAC in TiO_2_ Nanofilms to Maximize HER Performance

**Figure 4:**
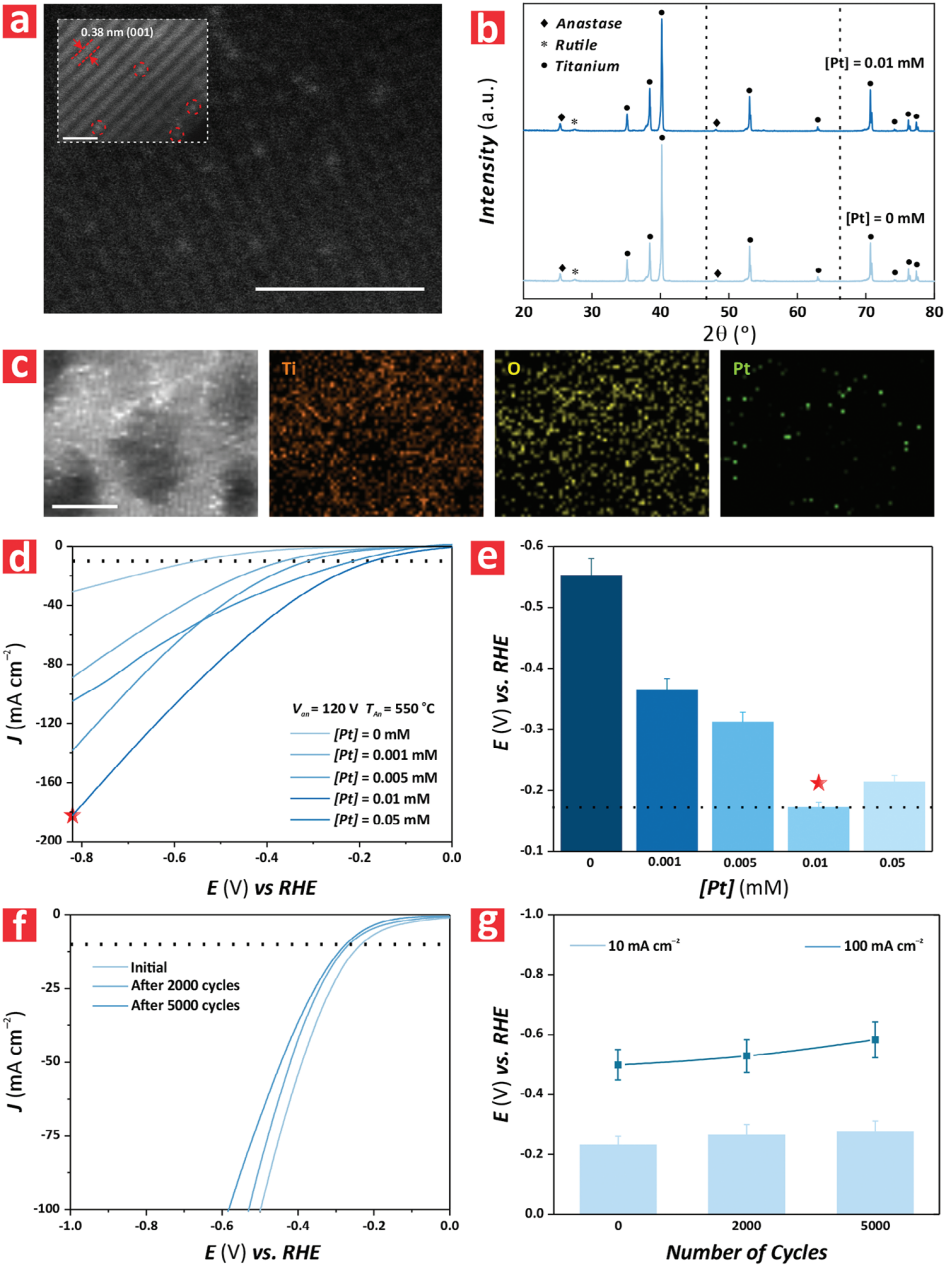
Chemical and electrochemical characterization of anodic TiO_2_ nanofilms modified with single‐atom Pt catalyst atoms at varying concentrations (*[Pt]* = 0, 0.001, 0.005, 0.01 and 0.05 m). a) HAADF‐TEM images of a representative anodic TiO_2_–120 nanofilms annealed at 550 °C modified with single‐atom Pt catalyst via dark deposition using a precursor with a concentration of *[Pt]* = 0.01 mm (scale bars = 2 nm and 1 nm (inset)). b) XRD spectra of TiO_2_–120 nanofilms annealed at *T_An_* 550 °C modified with single‐atom Pt catalyst via dark deposition using a precursor with a concentration of *[Pt]* = 0 and 0.01 mm (legends indicating the characteristic peaks of anatase, rutile and titanium and dotted lines denote expected positions of Pt nanoparticles). c) HAADF‐TEM images and EDX element mappings of TiO_2_–120 nanofilms annealed at *T_An_* 550 °C functionalized with single‐atom Pt catalyst via dark deposition using a precursor with a concentration of *[Pt]* = 0.01 mm. d) Linear sweep voltammograms of TiO_2_ nanofilms modified with single‐atom Pt catalyst atoms at varying concentrations (*[Pt]* = 0, 0.001, 0.005, 0.01 and 0.05 m) under varying overpotential (*E*) from −0.82 to 0.0 V versus RHE at a rate of 0.005 V s^–1^ in 1 m KOH. e) Overpotential values versus RHE measured in TiO_2_–120 nanofilms annealed at *T_An_* 550 °C functionalized with single‐atom Pt catalyst via dark deposition using a precursor with a concentration of *[Pt]* = 0, 0.001, 0.005, 0.01 and 0.05 mm at *η*_10_ in 1 m KOH (NB: data are presented as mean ± SD of a sample size of *n* ≥ 3 independent measurements). f) LSV stability assessment of anodic TiO_2_–120 nanofilms annealed at 550 °C modified with single‐atom Pt catalyst at a concentration of *[Pt]* = 0.01 m during 5000 HER cycles in 1 m KOH. g) Overpotential values versus RHE measured in TiO_2_–120 nanofilms annealed at *T_An_* 550 °C functionalized with single‐atom Pt catalyst at a concentration of *[Pt]* = 0.01 m after 0, 2000 and 5000 HER cycles at *η*_10_ and *η*_100_ in 1 m KOH.

Achieving an optimal distribution of SAC atoms within a host platform material and preventing agglomeration are factors of paramount importance for the realization of high‐performance SAC systems for HER.^[^ ^42^ ^]^ To this end, the mobility of SACs must be suppressed to stabilize SACs within the host material structure. Of all approaches, trapping SACs within atomic lattice defects is an effective approach to locally stabilizing SACs and ensuring their catalytic reactivity, which might be otherwise disrupted by diffusion and agglomeration.^[^ ^20^ ^]^ Our results indicated that thermal annealing at 550 °C is an effective approach to increasing reactive sites in the crystal lattice of anodic TiO_2_ nanofilms. Motivated by this, we decided to precisely modify the structure of these model films with single atom Pt catalyst through a dark deposition process.^[^ ^22^, ^43^ ^]^ **Figure** **4**a illustrates representative high‐angle annular dark‐field scanning transmission electron microscopy (HAADF‐STEM) images of SAC Pt on anodic TiO_2_–120 nanofilms annealed at 550 °C and subjected to dark deposition in 0.01 mm H_2_PtCl_6_ precursor solution for 24 h. Analysis of HAADF‐STEM images revealed that the Pt atoms were mostly present as individual SACs and as multimers (i.e., dimers and trimers), with no apparent atomic cluster formation. A magnified HAADF‐STEM image view (Figure 4a, inset) clearly showed the anatase crystal structure of the TiO_2_ nanofilms with a spacing of ≈0.38 nm generated upon the thermal annealing process. Figures S14 and S15 (Supporting Information) further demonstrate that most Pt atoms were trapped as isolated single atoms within the structure of the host TiO_2_ nanofilm platform, with only a few multimers being identified in the images. Figure 4b depicts the XRD spectra of an as‐produced TiO_2_–120 nanofilm annealed at 550 °C and its Pt‐functionalized counterpart after dark deposition exposure to a solution with *[Pt]* = 0.01 mm. It was apparent from these spectra that the SAC Pt‐modified TiO_2_–120 nanofilm did not feature the characteristic diffraction peaks of Pt nanoparticles at 2*θ* = 47.4° and 67.1° when compared to the reference as‐produced TiO_2_ nanofilm.^[^ ^44^ ^]^ The distribution and concentration of Pt atoms were further characterized by transmission electron microscopy energy‐dispersive X‐ray (TEM‐EDX) (Figure 4c). From these images, it was possible to identify that the composition of the host TiO_2_ nanofilms was limited to Ti, O, and Pt atoms. The latter element was in the form of single atoms, which were perfectly distributed across the crystalline structure of the host material and no Pt‐aggregates or Pt nanoparticles were observed. The complementary EDX analysis shown in Figures S14 and S15 (Supporting Information) further demonstrated that SAC Pt was evenly distributed across the TiO_2_ nanofilms. Figure 4e summarizes the HER performance assessment of model TiO_2_–120 nanofilms at *T*_An_ = 550 °C, the structure of which was modified with SAC Pt via dark deposition in distinct concentrations of H_2_PtCl_6_ precursor solution (i.e., *[Pt]* = 0, 0.001, 0.005, 0.01 and 0.05 mm). The value of *J* at *E* = −0.82 V versus RHE was measured to be −30.6 ± 0.3, −88.7 ± 0.6, −138.7 ± 1.6, −182.6 ± 2.3 and −104.7 ± 1.6 mA cm^−2^ for TiO_2_–120 nanofilms at *[Pt]* = 0, 0.001, 0.005, 0.01 and 0.05 mm, respectively. It was apparent from these results that the best performing TiO_2_–120 nanofilms were modified with *[Pt]* = 0.01 mm since it achieved the highest current density at the lowest overpotential. The analysis of *E* at *η*_10_ shown in Figure 4e indicated that the overpotential of TiO_2_–120 nanofilms functionalized with *[Pt]* = 0.01 mm was the lowest of all SAC‐modified TiO_2_–120 nanofilms assessed, with a value of −0.17 ± 0.01 V.

**Figure 5:**
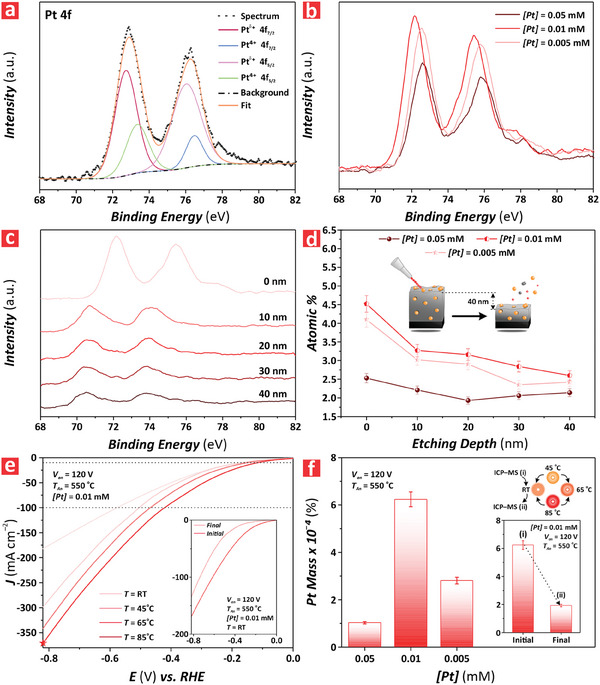
Chemical and electrochemical characterization of anodic TiO_2_ nanofilms modified with single‐atom Pt catalyst atoms at varying precursor concentrations (i.e., *[Pt]* = 0.005, 0.01, and 0.05 m) and reaction temperature (i.e., *T* = 25, 45, 65 and 85 °C). a) XPS spectra of Pt 4f of TiO_2_–120 nanofilms annealed at *T_An_* 550 °C and modified with single‐atom Pt catalyst via dark deposition using a precursor concentration of *[Pt]* = 0.01 mm. b) In‐depth XPS spectra profile of Pt 4f of a model TiO_2_–120 nanofilm annealed at *T_An_* 550 °C and modified with single‐atom Pt catalyst via dark deposition using a precursor with a concentration of *[Pt]* = 0.01 mm at distinct etching depths (i.e., 0, 10, 20, 30 and 40 nm). c) XPS spectra of Pt 4f of TiO_2_–120 nanofilms annealed at *T_An_* 550 °C and modified with single‐atom Pt catalyst via dark deposition using distinct precursor concentration (i.e., *[Pt]* = 0.005, 0.01 and 0.05 mm). d) An in‐depth profile of Pt atomic percentage at distinct etching depths, from 0 to 40 nm, for model TiO_2_–120 nanofilms annealed at *T_An_* 550 °C and modified with single‐atom Pt catalyst via dark deposition using distinct precursor concentration (i.e., *[Pt]* = 0.005, 0.01 and 0.05 mm) (NB: data are presented as mean ± SD of a sample size of *n* ≥ 3 independent measurements). e) Linear sweep voltammograms of TiO_2_–120 nanofilms modified with single‐atom Pt catalyst atoms at varying reaction temperatures (i.e., *T* = 25, 45, 65, and 85 °C) under varying overpotential (*E*), from −0.82 to 0.0 V versus RHE, at a rate of 0.005 V s^–1^ in 1 m KOH, with inset showing linear sweep voltammograms before and after HER. f) ICP–MS analysis of TiO_2_ nanofilms modified with single‐atom Pt catalyst atoms at varying concentrations (i.e., *[Pt]* = 0.005, 0.01 and 0.05 mm) before and after (inset) HER (NB: data are presented as mean ± SD of a sample size of *n* ≥ 3 independent measurements).

It was also possible to discern a direct correlation between the concentration of SAC precursor and HER efficiency, where *E* decreased with *[Pt]* until reaching its minimum at *[Pt]* = 0.01 mm. We performed a set of complementary experiments in which the HER performance of a model TiO_2_–120 nanofilms annealed at 550 °C and modified with Pt atoms using a precursor concentration of *[Pt]* = 0.01 mm was assessed over an extended number of cycles (i.e., 0, 2000, and 5000 HER cycles). To evaluate the performance of the SA‐modified TiO_2_–120 nanofilms, we quantified the overpotential (*E* vs *RHE*) at a current density of 10 and 100 mA cm^−2^ (i.e., *η*_10_ and *η*_100_). These results are summarized in Figure 4f,g. It was found that the overpotential of the photoelectrocatalyst increases slightly with the number of cycles, from −0.23 ± 0.02 to −0.28 ± 0.03 V at *η*_10_, and from −0.50 ± 0.05 to −0.58 ± 0.06 V at *η*_100_. The change in overpotential was attributed to the leaching of Pt atoms into the 1 m KOH electrolyte during multiple HER cycles. However, it is worth noting that the system stabilized after 2000 cycles, with a considerably reduced loss of performance with increasing cycles beyond this point, especially at *η*_10_. This would indicate that some loosely fixed Pt atoms on the surface of the nanofilms were released during the initial HER cycles but, after this, the system became almost stable at *η*_10_. Another interesting observation is that the performance deterioration with HER cycles is more substantial at higher current density, with an overall loss of overpotential of ≈1.15 and ≈4% after 5000 cycles at *η*_10_ and *η*_100_, respectively. Beyond this point, there was a slight increment in *E* at *[Pt]* = 0.05 mm. This trend might be attributed to the efficient accommodation of Pt atoms within the crystal lattice structure of the annealed TiO_2_–120 nanofilms as the concentration of precursor increased from 0 to 0.01 mm. However, there was a critical point when the precursor solution was 0.05 mm at which the crystal lattice was saturated with Pt atoms. This in turn led to the formation of clusters by free Pt atoms, resulting in the observed worsening of HER performance (i.e., *E* = −0.22 V versus RHE for *[Pt]* = 0.05 mm). Figure S16 (Supporting Information) shows a top view FEG‐SEM image of a representative TiO_2_–120 nanofilm annealed at 550 °C and modified with SAC at *[Pt]* = 0.01 mm after 5000 HER cycles. This analysis indicates that the nanofilms do not undergo any apparent change in morphology or structure after the multiple HER cycles, which indicates good structural stability as demonstrated by the electrochemical measurements. Figure S17 (Supporting Information) shows a comparison of the real‐time *J*–*t* response of as‐produced and Pt‐functionalized TiO_2_ nanofilms under ON and OFF states, where the latter was modified with *[Pt]* = 0.05 mm. It is apparent that the presence of single Pt atoms within the structure of the anodic film increased the photoanodic current significantly (i.e., ≈19.7%). Figures S18 and S19 (Supporting Information) illustrate the electrocatalytic performance of annealed TiO_2_ nanofilms under dark conditions, and the overpotentials at *η*_10_ under ON and OFF illumination states, respectively. These analyses indicated that there was a decreasing trend in *E* with *[Pt]* for the OFF‐illumination state, which followed the same pattern as that seen in the ON state. It was also found that the value of Δ*E* = *E_ON_*–*E_OFF_* increased slightly with *[Pt]*, with an average value of 0.023 ± 0.002 V at *[Pt]* = 0 mm and 0.024 ± 0.002 V at *[Pt]* = 0.05 mm. To corroborate our hypothesis about the origin of HER performance deterioration when the concentration of precursor increased from 0.01 to 0.05 mm, the structure of TiO_2_–120 nanofilms modified with *[Pt]* = 0.05 mm was analyzed by HAADF‐STEM.^[^ ^45^ ^]^ Figure S20 (Supporting Information) shows that the vast majority of Pt present in these films was in the form of single atoms. However, more multimers and the formation of clusters can be clearly observed when compared to the TiO_2_–120 nanofilms modified with *[Pt]* = 0.01 mm. This would justify the worsening in HER catalytic activity attributed to the increased formation of clusters and a reduction in the active specific area of Pt atoms. To further verify this result, **Figure** **5**a shows the XPS spectrum for the Pt 4f regions of a TiO_2_ nanofilm after dark deposition of Pt from a precursor solution at *[Pt]* = 0.01 mm. The characteristic peak position for Pt 4f_7/2_ was located at a binding energy of 72.80 eV, which corresponded to single atom Pt^δ+^ with δ ≈2.^[^ ^22^, ^46^ ^]^ Figure 5b shows the XPS spectra of the Pt 4f regions of TiO_2_ nanofilms modified with SAC via dark deposition using three different concentrations of Pt precursor (i.e., *[Pt]* = 0.005, 0.01 and 0.05 mm). This analysis indicated that the intensity of the Pt 4f peaks of TiO_2_ nanofilms increased when the concentration of precursor was increased from 0.005 to 0.01 mm. This was followed by a sharp decrease in intensity when *[Pt]* was further increased to 0.05 mm. At this point, the Pt 4f peaks reached their lowest intensity. This result suggested that at that concentration of precursor, large nanoclusters and nanoparticles formed and aggregated across the surface of the TiO_2_ nanofilms, preventing free Pt atoms from diffusing into deeper regions of the bulk TiO_2_ nanofilms and reducing the overall content of Pt in the TiO_2_ nanofilms.^[^ ^47^ ^]^ However, this analysis only provided information about the nature of Pt atoms on the surface of the photoelectrocatalyst. To gain further insights into the formation of Pt clusters and how Pt atoms diffused within the thickness of the TiO_2_ nanofilms, we performed an in‐depth selective elemental analysis combining XPS with Ar^+^ milling on TiO_2_ nanofilms modified with SAC Pt atoms from precursor solutions at *[Pt]* = 0.005, 0.01 and 0.05 mm. XPS spectra of these films were collected after 10 nm etching steps from the surface of the film to create an in‐depth profile of Pt distribution, from 0 to 40 nm (Figure 5c). Figure S21 (Supporting Information) shows the raw XPS spectra of the etched TiO_2_ nanofilms at distinct etching depths (i.e., 0, 10, 20, 30, and 40 nm). It is apparent that the intensity associated with Pt 4f in the TiO_2_ nanofilms modified by precursor solutions of *[Pt]* = 0.005 and 0.01 mm decreased with the etching depth. Figure 5d represents the correlation between the atomic percentage of Pt and the etching depth for TiO_2_ nanofilms at *[Pt]* = 0.005, 0.01, and 0.05 mm. It was found that the highest concentration of Pt atoms on the surface of the film was achieved by TiO_2_ nanofilms functionalized with *[Pt]* = 0.01 mm. Intriguingly, the concentration of Pt in the nanofilm functionalized with the precursor solution with the highest concentration of Pt (i.e., *[Pt]* = 0.05 mm) was the lowest at any depth. The in‐depth profile also revealed that for the case of TiO_2_ nanofilms functionalized with the precursor solutions *[Pt]* = 0.005 and 0.01 mm, there was a progressive decrease in Pt with the etching depth. Conversely, at *[Pt]* = 0.05 m it was found that the concentration of SAC was the lowest along all the in‐depth profiles and remained statistically constant throughout the bulk of the semiconductor.

This result was further confirmed by inductively coupled plasma mass spectrometry (ICP–MS) analysis (vide infra) and would further demonstrate the formation of clusters of Pt on the external surface of the TiO_2_ nanofilms functionalized at *[Pt]* = 0.05 mm and the inefficient intercalation of SAC in the semiconductor crystal lattice structure.

### 2.4 Tuning of Reaction Temperature in TiO_2_ Nanofilms to Maximize HER Performance

Photothermal catalysis has been devised as an efficient path to increasing the rate of catalytic hydrogen production through thermochemistry effects.^[^ ^48^ ^]^ Motivated by this, we analyzed the HER performance of a model TiO_2_ nanofilm with optimal thickness, crystallographic phase, and SAC functionalization under a range of reaction temperatures. Figure 5e illustrates the HER performance assessment of model TiO_2_–120 nanofilms annealed at *T*_An_ = 550 °C and modified with SACs at *[Pt]* = 0.01 mm when the reaction was performed at distinct electrolyte reaction temperatures, from 25 to 85 °C. The current density measured at the lowest overpotential (i.e., *E* = −0.82 V vs RHE) was found to be −169.4 ± 8.5, −284.3 ± 14.2, −325.6 ± 16.3 and −355.7 ± 17.8 mA cm^−2^ at 25, 45, 65 and 85 °C, respectively. Therefore, the best performance in HER was achieved at a reaction temperature of 85 °C. Figure S22 (Supporting Information) summarizes the overpotential values at *η*_10_ estimated from Figure 5e, where *E* was measured to be −0.552 ± 0.028, −0.183 ± 0.009, −0.168 ± 0.008 and −0.135 ± 0.007 V versus RHE at 25, 45, 65 and 85 °C, respectively. Figures S23 and S24 (Supporting Information) show the electrocatalytic performance at different reaction temperatures under non‐illuminated conditions, and a comparison of overpotentials at *η*_10_ under ON and OFF illumination states, respectively. Figures S25 and S26 (Supporting Information) depict the electrocatalytic performance at different reaction temperatures under non‐illuminated conditions, and a comparison of overpotentials at *η*_100_ under ON and OFF illumination states, respectively. Both illuminated and non‐illuminated HER performance of TiO_2_ nanofilms showed comparable trends, where there was a progressive decrease in *E* with increasing reaction temperature, from 25 to 85 °C. However, it is worth noting that there was a deterioration in the performance of the photoelectrocatalyst film after multiple HER cycles at increasing reaction temperatures. A model SAC TiO_2_ nanofilm was subjected to sequential HER reactions at electrolyte temperatures of 25, 45, 65, and 85 °C, and then assessed again at a reaction temperature of 25 °C. The model photoelectrocatalyst underwent an increment in overpotential after the cyclic HER treatment (see inset in Figure 5e; Figure S27, Supporting Information). The deactivation temperature of Pt—a noble metal catalysts is >600 °C.^[^ ^49^ ^]^ Because the reaction temperature is well below this value, Pt atoms embedded into the TiO_2_ nanofilms should remain active. However, it has been reported that the electrolyte reaction temperature can lead to agglomeration and sintering of SACs immobilized in the host photoelectrocatalyst, which in turn negatively impacts the catalytic activity by decreasing active sites within the host material.^[^ ^50^ ^]^ Another potential negative effect impacting the catalytic activity of model TiO_2_–120 nanofilms upon multiple HER cycles in the reactant solution of increasing temperature might be attributed to the loss of SA Pt into the electrolyte via leaching. Kinoshita et al determined that the loss of SAC Pt atoms during HER is reliant on the reaction temperature, the range of voltage, and the number of HER cycles.^[^ ^51^ ^]^ Under the conditions of the study, both the applied voltage bias range and number of HER cycles remained constant. Therefore, the loss of Pt atoms observed might be primarily associated with thermal effects from the electrolyte temperature, which is in good agreement with previous studies.^[^ ^52^ ^]^ To elucidate this, we used inductively coupled plasma mass spectrometry (ICP–MS) to analyze the mass difference of Pt content in TiO_2_ nanofilms before and after HER cycles at distinct electrolyte temperatures. Figure 5f shows the Pt weight percentage content in TiO_2_ nanofilms functionalized with different concentrations of precursor (i.e., *[Pt]* = 0.005, 0.01, and 0.05 mm) before HER. The weight percentage of Pt in TiO_2_ nanofilms functionalized with *[Pt]* = 0.005, 0.01, and 0.05 mm established via ICP–MS was determined to be 2.81 ± 0.14 × 10^−4^, 6.24 ± 0.31 × 10^−4^ and 1.03 ± 0.05 × 10^−4^%, respectively (Tables S1 and S2, Supporting Information). This analysis was in good agreement with the lower intensity of Pt 4f and atomic percentages of TiO_2_ nanofilms determined by XPS (Figure 5c,d). The analysis of the Pt percentage before and after HER reaction, labeled as “initial” and “final”, respectively, for the TiO_2_ nanofilms modified with *[Pt]* = 0.01 mm at a reactant electrolyte temperature of 25 °C shown in Figure 5f (inset) revealed that there was a three‐fold decrement in Pt, from 6.24 ± 0.31 × 10^−4^ to 1.93 ± 0.10 × 10^−4^%, upon the cyclic HER at increasing temperature (i.e., 25, 45, 65, 85, and 25 °C). Tables S2 and S3 (Supporting Information) show detailed information obtained from ICP–MS analysis, including calculation formulas.

### 2.5 Mechanistic Framework for Designing High‐Performance Single‐Atom Nanofilm HER Photoelectrocatalysts

**Figure 6:**
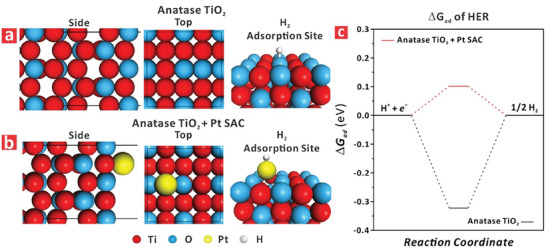
Mechanistic description of crystallographic phase and single‐atom Pt catalyst inclusion effect of TiO_2_ nanofilms on HER performance in terms of Gibbs free energy required for hydrogen adsorption estimated from density‐functional theory calculations (DFT). a) Side and top view of the atomic structure of anatase phase TiO_2_ with hydrogen adsorption site of Ti. b) Side and top view of the atomic structure of anatase phase TiO_2_ with deposited single‐atom Pt and hydrogen adsorption site of Pt. c) Reaction profile for H binding and H_2_ evolution on as‐produced and single‐atom Pt catalyst‐modified anatase TiO_2_.

It has been empirically demonstrated that the rational design of film thickness, morphology, crystallographic phase, number of single‐atom Pt catalyst inclusions, and the electrolyte temperature can be synergistically harnessed to maximize the generation of electron–hole pairs that contribute to the output current density at a reduced overpotential to drive HER in model TiO_2_ nanofilms. To provide a mechanistic framework that supports our experimental observations, we performed DFT calculations to analyze the absorption behavior of hydrogen on anatase phase in TiO_2_ modified with single‐atom Pt for HER. **Figure** **6**a shows the top and side view atomic structures of anatase crystals with details of the hydrogen adsorption site on Ti atoms. Figure 6b shows the top and side view of atomic structures of the counterpart anatase crystals modified with single‐atom Pt inserted in an oxygen vacancy within the TiO_2_ lattice (top view in Figure 6a). Upon exposure to the electrolyte, hydrogen was adsorbed onto the Pt atom inclusion as depicted in Figure 6b. In this analysis, the H binding energy to anatase TiO_2_ and to its SAC Pt‐modified counterpart was calculated. Figure 6c compares the Gibbs free energy of H adsorption (Δ*G_ad_*) in as‐produced and SA Pt‐modified anatase TiO_2_ phases. These values were calculated to be −0.32 and 0.10 eV, respectively. A value of Δ*G_ad_* in the proximity of 0.00 eV would indicate an optimal reaction condition for hydrogen adsorption since less energy is required for this process to occur. Hence, SA Pt‐modified anatase TiO_2_ provided a better reaction platform than as‐produced anatase TiO_2_, with almost a two‐fold reduction in H adsorption‐free energy with an optimum value close to 0.00 eV.^[^ ^53^ ^]^

The simulation data corroborated our experimental findings that anatase phase modified with Pt atoms co‐located in oxygen vacancies achieved higher catalytic activity than that of its as‐produced analog in terms of hydrogen adsorption for HER. Our theoretical analysis also corroborated that the inclusion of a single‐atom Pt catalyst in TiO_2_ nanofilms provided a suitable scheme to boost HER performance. However, maximizing HER performance by adding inclusions of SA Pt as co‐catalysts requires careful selection of the concentration of Pt precursor to avoid the formation of clusters, which were found to have a detrimental effect. To further corroborate our results, we simulated the effect of the electrolyte temperature on the HER reaction rate. Table S4 (Supporting Information) compiles the Gibbs free energy of hydrogen adsorption at the reaction temperatures of 25, 45, 65, and 85 °C for as‐produced and SA Pt‐modified anatase phases for HER. It was found that the required energy for hydrogen adsorption on anatase TiO_2_ with single‐atom Pt catalyst inclusions decreased from −0.32 to −0.28 eV when the electrolyte temperature was increased from 25 to 85 °C, respectively, which was also in good agreement with our experimental observations.

## 3 Conclusions

In summary, this study provides a comprehensive methodology with critical insights into the rational design of thin film single‐atom photoelectrocatalysts for hydrogen evolution reaction, using anodic titanium dioxide nanofilms as a model host material. It has been determined that a careful design consideration of film thickness and morphology, crystallographic phase of TiO_2_, quantification of single‐atom Pt catalyst inclusions, and reaction temperature can be synergistically harnessed to boost HER performance by more than one order of magnitude. Our study has determined that the thinner the anodic film is the better the HER performance. However, the generation of pits on the surface of thicker films at high anodization voltage enhanced the efficiency of the reaction by increasing the effective area, despite the increasing overall thickness of the film. The crystallographic phase of TiO_2_ in the nanofilm determines HER efficiency by increasing the reactive facets in the crystal lattice, where anatase phase form produced at 550 °C was found to be the best‐performing structure. The number of single‐atom Pt catalyst inclusions in the lattice of TiO_2_ requires a precise distribution of atoms to overcome agglomeration and the formation of clusters. A precursor concentration of 0.001 m to modify the structure of anatase TiO_2_ nanofilms through dark deposition electrolyte provided the best performing HER efficiency. The temperature of the reactant solution is also another critical factor in maximizing HER performance harnessing thermochemical effects. An electrolyte temperature of 85 °C made it possible to enhance HER output. However, the extended use of the photoelectrocatalyst in these conditions worsens with HER cycles due to the leaching of SACs into the electrolyte. The findings reported in this study pave the way for better design of single‐atom photoelectrocalysts, which could be extended to other systems. Key fundamental questions also remain about the use of other SAC systems, including synergies between dual single‐atom species. These new design strategies may provide new avenues for achieving highly efficient HER performances in fully scalable photoelectrocatalysts for real‐life applications.

## 4 Experimental Section

### TiO_2_ Nanofilm Fabrication

TiO_2_ nanofilms were grown on Ti foils (i.e., 0.2 mm thick, Goodfellow, 99.6+ % purity) by electrochemical anodization, using aqueous 0.5 m H_2_SO_4_ electrolyte (Sigma‐Aldrich, Australia). Ti foils were anodized at 20, 40, 60, 80, 100, and 120 V for 3 mins at room temperature. Before anodization, the Ti foils were cut into 1.5 × 1.5 cm chips, washed in EtOH and Milli–Q water and air dried. Annealed TiO_2_ nanofilms were treated in atmospheric conditions at different temperatures (i.e., RT, 350, 550, and 750 °C) for 1 h.

### Dark Deposition

Annealed TiO_2_ nanofilms were immersed in 10 mL MeOH (50 *v*%) solution containing varying concentrations of H_2_PtCl_6_∙6H_2_O precursor (Sigma–Aldrich, Australia). The reaction was performed in a reactor purged with Ar gas for 15 min to maintain a neutral atmosphere. After 24 h, the nanofilms were washed in EtOH, and Milli–Q water for 15 min each, and then air dried and stored in an inert atmosphere until further use.

### Structural and Chemical Characterization

The morphology and thickness of TiO_2_ nanofilms were characterized by field emission gun scanning electron microscopy (FEG‐SEM Quanta 450, FEI). The thickness of TiO_2_ thin films was determined from FEG‐SEM image analysis using ImageJ and by ellipsometry measurements. The crystal structures of as‐produced and annealed TiO_2_ nanofilms were characterized by X‐ray diffraction (XRD, Empyrean, using MDI Jade 9 software to identify lattice type. Raman spectroscopy was measured in a Raman confocal microscope (LabRAM HR Evolution, Horiba) with laser excitation at 532 nm. HAADF‐STEM and EDX mapping analyses were obtained by a high‐resolution transmission electron (FEI Titan Themis 80–200). Oxygen vacancies were measured by electron paramagnetic resonance (EPR, Bruker A300). The chemical composition profile of TiO_2_ nanofilms was obtained by X‐ray photoelectron spectroscopy (XPS, Thermo Fisher Scientific K‐Alpha). The mass fractions of Pt in TiO_2_ nanofilms were quantified by inductively coupled plasma mass spectrometry (ICP–MS, Agilent 720ES). The electronic band gap of model TiO_2_ nanofilms was determined from diffuse reflectance measurements performed in a Shimadzu UV 2600i spectrometer equipped with an integrating sphere.

### Photoelectrochemical Measurements

Photoelectrochemical HER measurements were characterized in a three‐electrode system with an aqueous 1 m potassium hydroxide (KOH; pH ≈ 14) solution as an electrolyte saturated with Ar gas, using an electrochemical station (CHI760E, CHI Instruments Inc.). The three‐electrode system consists of a reference electrode (Ag/AgCl), a counter electrode (Pt), and TiO_2_ nanofilms as the working electrode. The effective area of TiO_2_ nanofilms was 0.63 cm^2^ for all the electrochemical measurements. The scan rate of 5 mV s^–1^ was used for all linear sweep voltammetry measurements. All applied biases were converted into RHE. A 300 W xenon lamp (PLS‐SXE300, Beijing Perfectlight, 1.2 W cm^−2^) was used as a light source to simulate broad‐spectrum solar light. AC impedance was applied to analyze the properties of TiO_2_ nanofilms with distinct thicknesses in an electrochemical station in 1 m KOH. The frequency range for EIS measurements was from 0.1 MHz to 0.1 Hz with a small sinusoidal AC voltage of 5 mV. Chronoamperometry of the TiO_2_ nanofilms was performed at −0.06 V versus Ag/AgCl with 10 s intervals for the ON−OFF illumination modes.

### DFT Computations

All DFT calculations were conducted using the Vienna ab initio simulation package (VASP) with Perdew‐Burke‐Ernzerhop (PBE) exchange‐correlation and projector‐augmented wave (PAW) potential to describe the ionic cores.^[^ ^54^ ^]^ Anatase and rutile surface structure were calculated by setting a 450 eV plane‐wave cutoff energy and a Monkhorst‐Pack^[^ ^55^ ^]^ k‐point grid of 3 × 3 × 1. The selected anatase and rutile surface structures were (001) and (110), respectively (Table S5, Supporting Information). The DFT‐D3 was utilized to address Van der Waals interactions, where the convergence of the energy was set to be 1 × 10^−4^ eV and that of geometry optimization was set to be maximum force ≤ 0.03 eV A^–1^.^[^ ^56^ ^]^ The 3d orbital electrons of Ti were treated using GGA+U method of Dudarev et al, with an effective Hubbard field Coulomb interaction parameter (*U*′ = *U*−*J*).^[^ ^57^ ^]^ According to the literature, the choice of *U* = 4 eV for the surface structure of rutile and anatase types is confident.^[^ ^58^ ^]^ The Gibbs free energy (*G*_ad_) of hydrogen at the sites of Ti and Pt was calculated as in Equation 1:

(1) $$G_{\mathrm{ad}}=E_{\mathrm{ad}}+\mathrm{ZPE}+\int C_{P}\mathrm{dT}-\mathrm{TS}$$

where *E*_ad_ is the adsorption energy, *ZPE* is the zero‐point energy, ∫*C_P_ dT* is the enthalpic temperature correction, and TS is the entropic correction.

The adsorption energy (*E*_ad_) was calculated by Equation 2:

(2) $$E_{\mathrm{ad}}=E_{\mathrm{Tot}}\;-E_{\mathrm{Surf}}-\frac{1}{2}\mu_{H_{2}}$$

where *E*_Tot_ is the DFT total energy of the surface with adsorbate, *E*_Surf_ is the energy of the corresponding pristine surface and *µ*_H2_ is the chemical potential for H_2_.

The chemical potential for H_2_ ($\mu_{H_{2}}$) by varied temperature is calculated by Equation 3:

(3) $$\mu_{H_{2}}=\;E\left(H_{2}\right)+\mathrm{ZPE}\left(H_{2}\right)+\int C_{P}\mathrm{dT}\left(H_{2}\right)-\mathrm{TS}\left(H_{2}\right)$$

## Conflict of Interest

The authors declare no conflict of interest.

## Supporting information

Supporting Information

## Data Availability

The data that support the findings of this study are available from the corresponding author upon reasonable request.
