# Supplementary material for: Elucidating Synergies of Single‐Atom Catalysts in a Model Thin Film Photoelectrocatalyst to Maximize Hydrogen Evolution Reaction
Source: Adv Sci (Weinh). 2024 Sep 4;11(41):2407598. doi: 10.1002/advs.202407598 (PMC11538634; doi:10.1002/advs.202407598)
Supplement: Supplementary file 1 — Supporting Information [file ADVS-11-2407598-s001.pdf]

ADVANCED SCIENCE
Open Access

Supporting Information

for *Adv. Sci.*, DOI 10.1002/advs.202407598

Elucidating Synergies of Single-Atom Catalysts in a Model Thin Film Photoelectrocatalyst to Maximize Hydrogen Evolution Reaction

Zichu Zhao, Cheryl Suwen Law, Yanzhang Zhao, Jairo Alberto Baron Jaimez, Amin Talebian-Kiakalaieh, Haobo Li, Jingrun Ran, Yan Jiao, Andrew D. Abell and Abel Santos**

Supporting Information

Elucidating Synergies of Single-Atom Catalysts in a Model Thin Film Photoelectrocatalyst to Maximize Hydrogen Evolution Reaction

Zichu Zhao, Cheryl Suwen Law, Yanzhang Zhao, Jairo Alberto Baron Jaimez, Amin Talebian-Kiakalaieh, Haobo Li, Jingrun Ran, Yan Jiao, Andrew D. Abell, and Abel Santos**

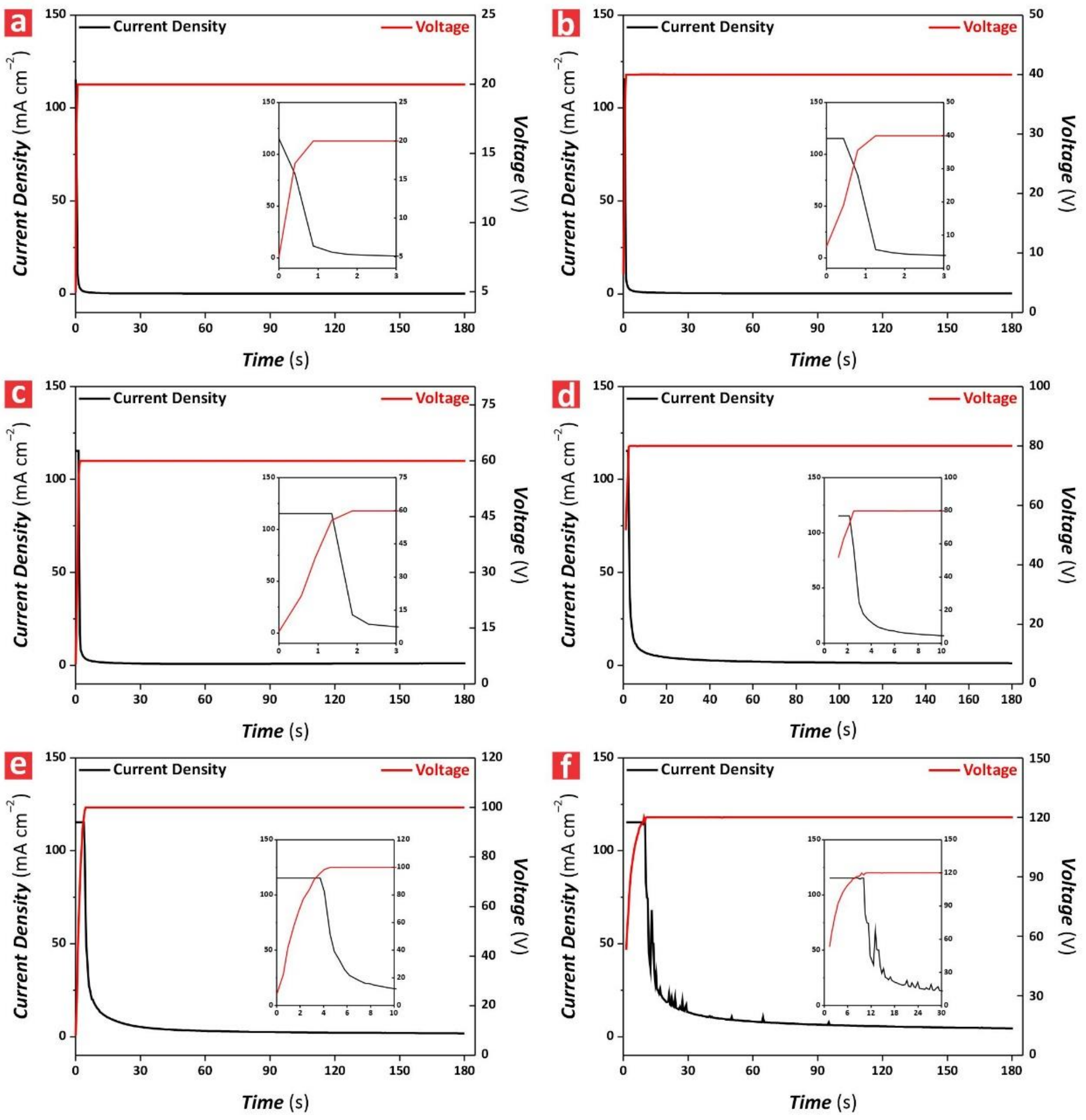

Figure S1. Anodization profiles used to produce TiO_2 nanofilms by potentiostatic anodization in 0.5 M H_2SO_4 electrolyte at distinct anodizing potentials (V_{an}): (a) TiO_2–20 at V_{an} = 20 V. (b) TiO_2–40 at V_{an} = 40 V. (c) TiO_2–60 at V_{an} = 60 V. (d) TiO_2–80 at V_{an} = 80 V. (e) TiO_2–100 at V_{an} = 100 V. (f) TiO_2–120 at V_{an} = 120 V.

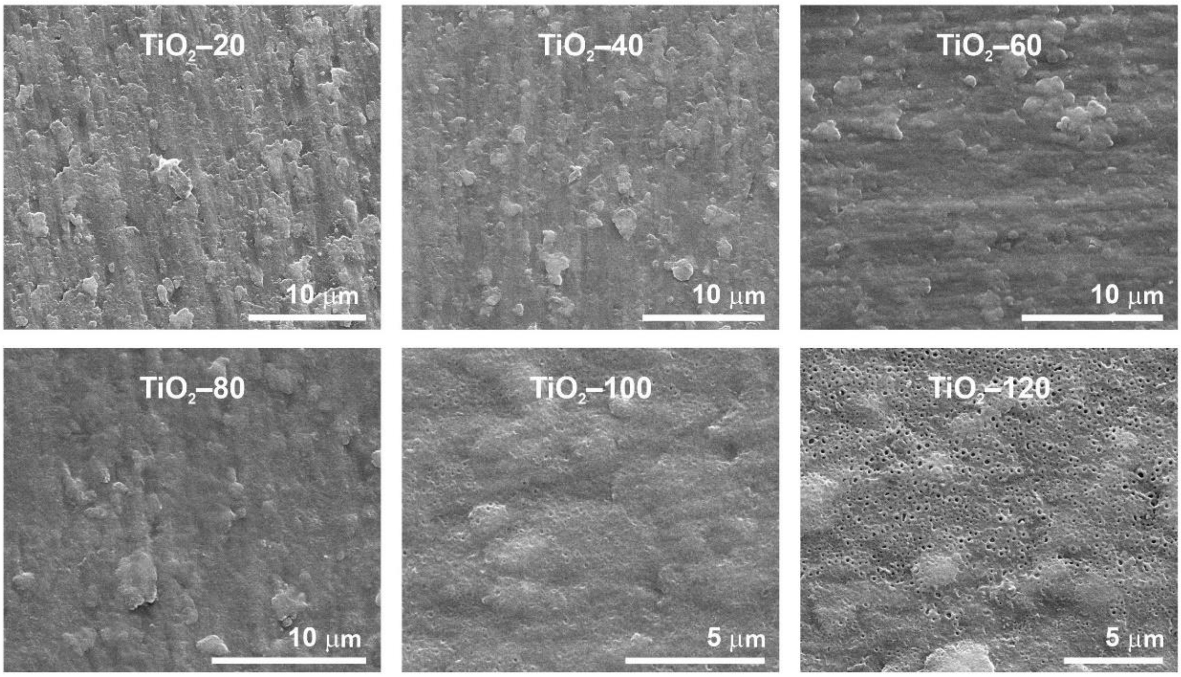

Figure S2. Top view of FEG-SEM images of TiO_2 nanofilms fabricated at distinct anodizing potentials: TiO_2–20 at V_{an} = 20 V; TiO_2–40 at V_{an} = 40 V; TiO_2–60 at V_{an} = 60 V; TiO_2–80 at V_{an} = 80 V; TiO_2–100 at V_{an} = 100 V; and TiO_2–120 at V_{an} = 120 V.

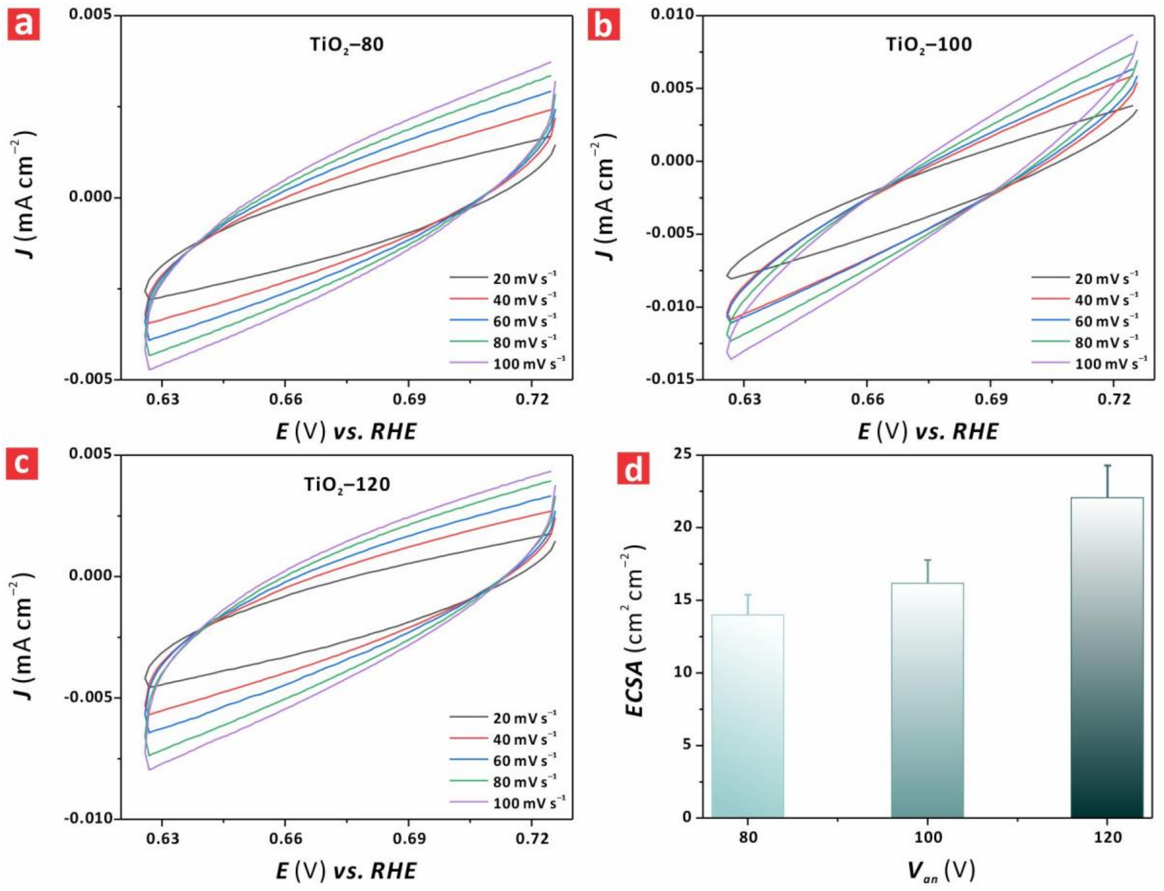

Figure S3. Cyclic voltammetry and estimation of electrochemical surface area (ECSA) of TiO_2 nanofilms fabricated at distinct anodizing potentials: TiO_2–80, TiO_2–100, and TiO_2–120 fabricated at V_{an} = 80, 100 and 120 V, respectively. (a) Cyclic voltammetry graph of TiO_2–80. (b) Cyclic voltammetry graph of TiO_2–100. (c) Cyclic voltammetry graph of TiO_2–120. (d) Electrochemical surface area of TiO_2–80, TiO_2–100, and TiO_2–120 nanofilms. (NB 1: data are presented as mean ± SD of a sample size of $n \geq 3$ independent measurements; NB 2: ECSA values were calculated by using the equation: ECSA = C_{dl} / C_s, where C_{dl} is the double-layer capacitance extracted from the cyclic voltammetry graphs and C_s is the specific capacitance (i.e., C_s = 0.86 μF/cm^{-2}[1])).

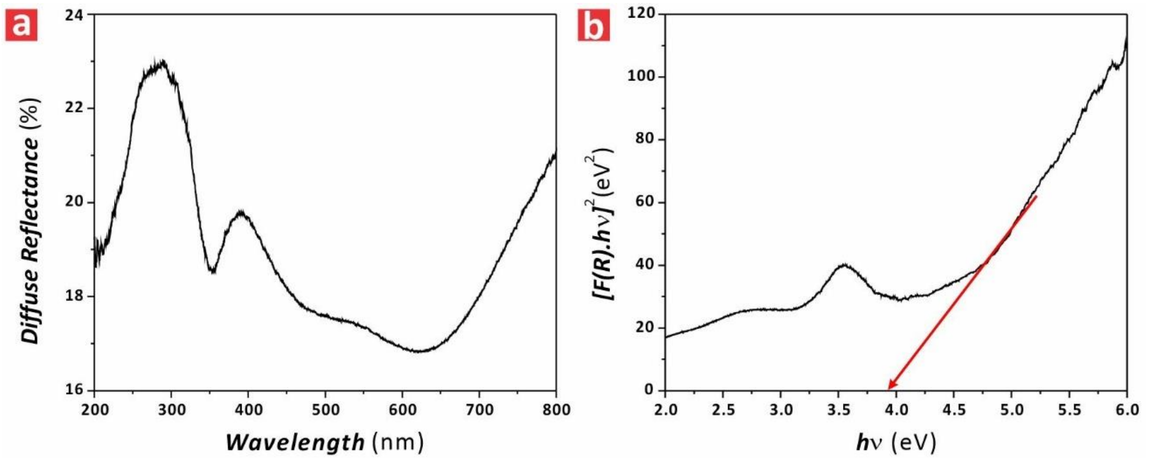

Figure S4. UV–Visible diffuse reflectance spectra and simulated Schottky diagrams of TiO_2–120 nanofilms produced at V_{an} = 120 V. (a) UV–Visible diffuse reflectance spectra of TiO_2–120 nanofilm. (b) Schottky diagram with red arrow indicating the energy band gap of the TiO_2–120 nanofilm.

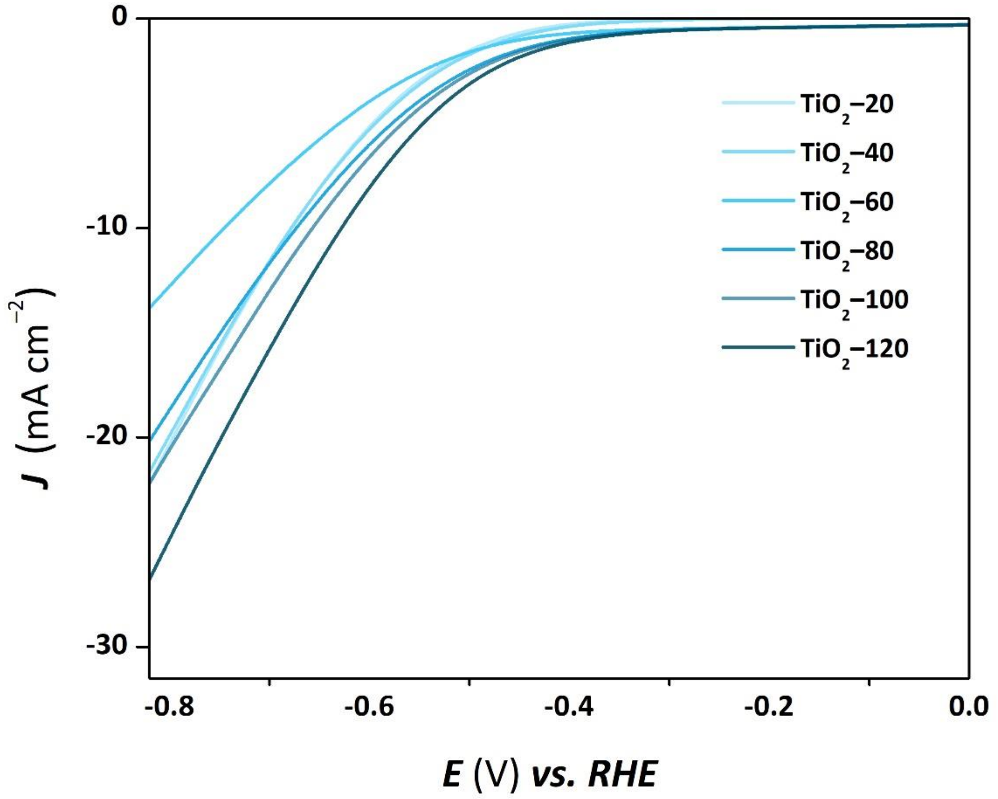

Figure S5. Linear sweep voltammograms of TiO_2 nanofilms fabricated at distinct anodizing potentials (i.e., TiO_2–20 at V_{an} = 20 V; TiO_2–40 at V_{an} = 40 V; TiO_2–60 at V_{an} = 60 V; TiO_2–80 at V_{an} = 80 V; TiO_2–100 at V_{an} = 100 V; and TiO_2–120 at V_{an} = 120 V) under non-illumination conditions and varying overpotential (*E*), from –0.82 to 0.0 V *vs* RHE, at a rate of 0.005 V s^{-1} in 1 M KOH.

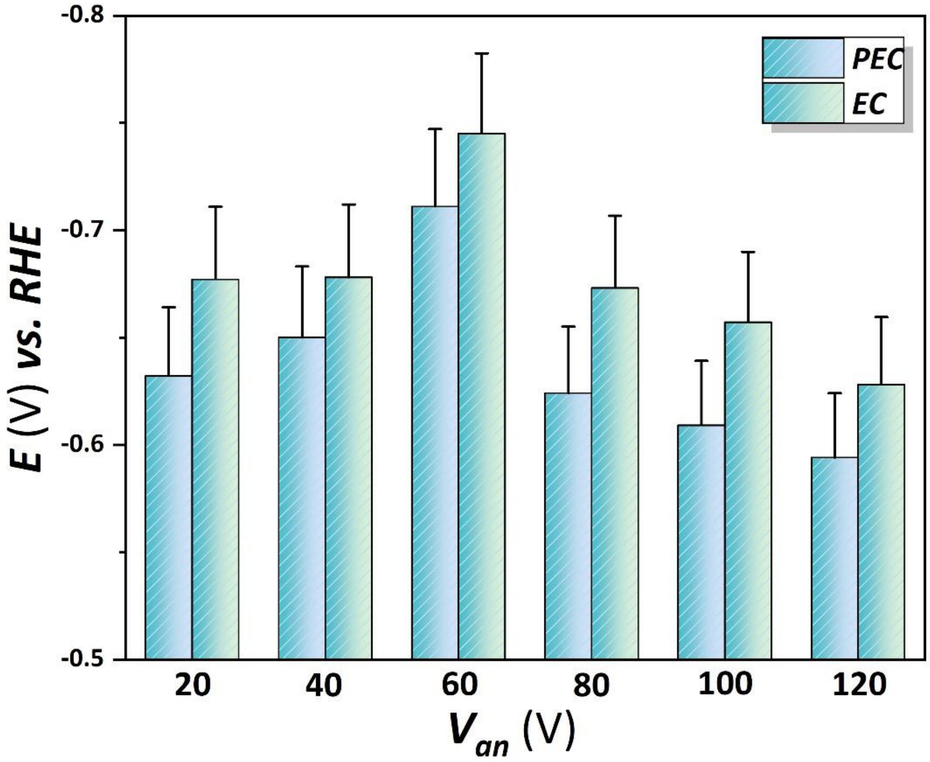

Figure S6. Overpotential values *vs* RHE measured in TiO_2 nanofilms fabricated at distinct anodizing potentials (i.e., TiO_2–20 at V_{an} = 20 V; TiO_2–40 at V_{an} = 40 V; TiO_2–60 at V_{an} = 60 V; TiO_2–80 at V_{an} = 80 V; TiO_2–100 at V_{an} = 100 V; and TiO_2–120 at V_{an} = 120 V) under illumination and non-illumination conditions to deliver a current density of J = 10 mA cm^{-2} in 1 M KOH (i.e., performance metric for HER) (NB: data are presented as mean ± SD of a sample size of $n \geq 3$ independent measurements).

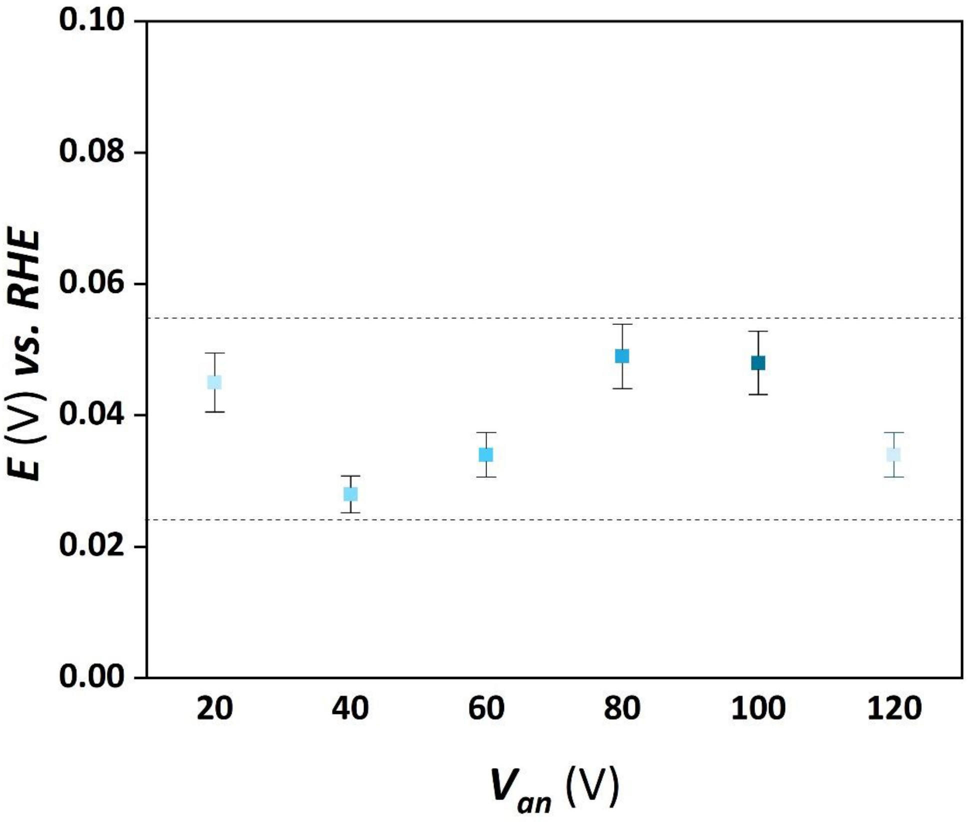

Figure S7. Differential photocurrent between illuminated (ON) and non-illuminated (OFF) conditions for TiO_2 nanofilms fabricated at distinct anodizing potentials (i.e., TiO_2–20 at V_{an} = 20 V; TiO_2–40 at V_{an} = 40 V; TiO_2–60 at V_{an} = 60 V; TiO_2–80 at V_{an} = 80 V; TiO_2–100 at V_{an} = 100 V; and TiO_2–120 at V_{an} = 120 V) (NB: data are presented as mean ± SD of a sample size of $n \geq 3$ independent measurements).

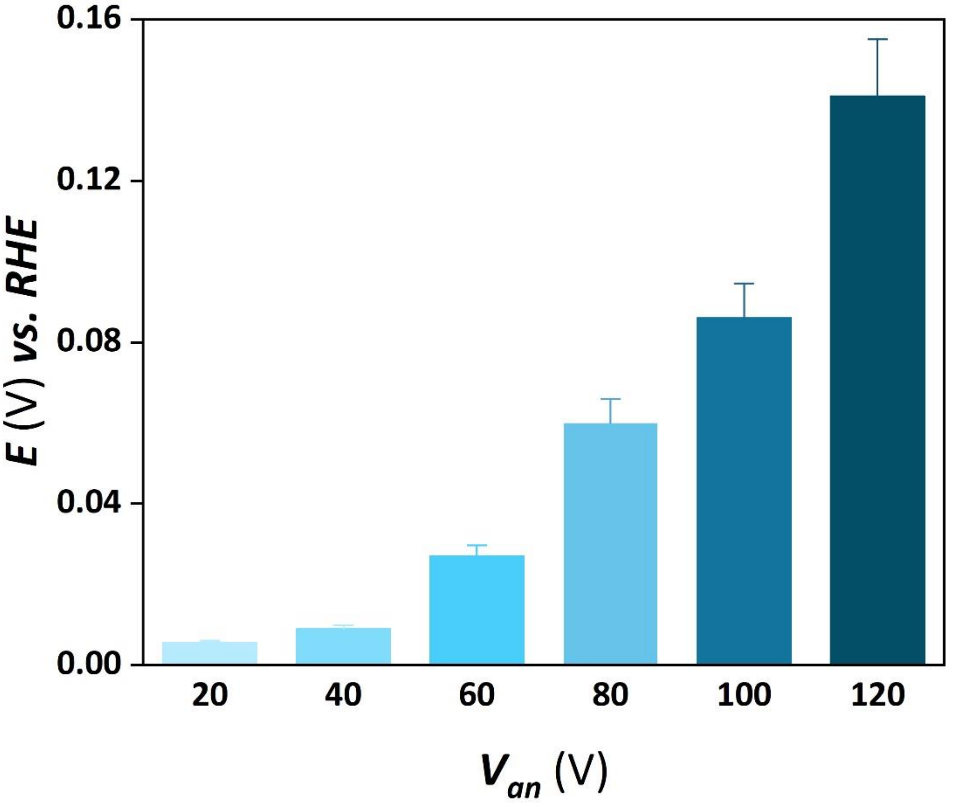

Figure S8. Increment light response current density of TiO_2 nanofilms fabricated at distinct anodizing potentials (i.e., TiO_2–20 at V_{an} = 20 V; TiO_2–40 at V_{an} = 40 V; TiO_2–60 at V_{an} = 60 V; TiO_2–80 at V_{an} = 80 V; TiO_2–100 at V_{an} = 100 V; and TiO_2–120 at V_{an} = 120 V) (NB: data are presented as mean ± SD of a sample size of $n \geq 3$ independent measurements).

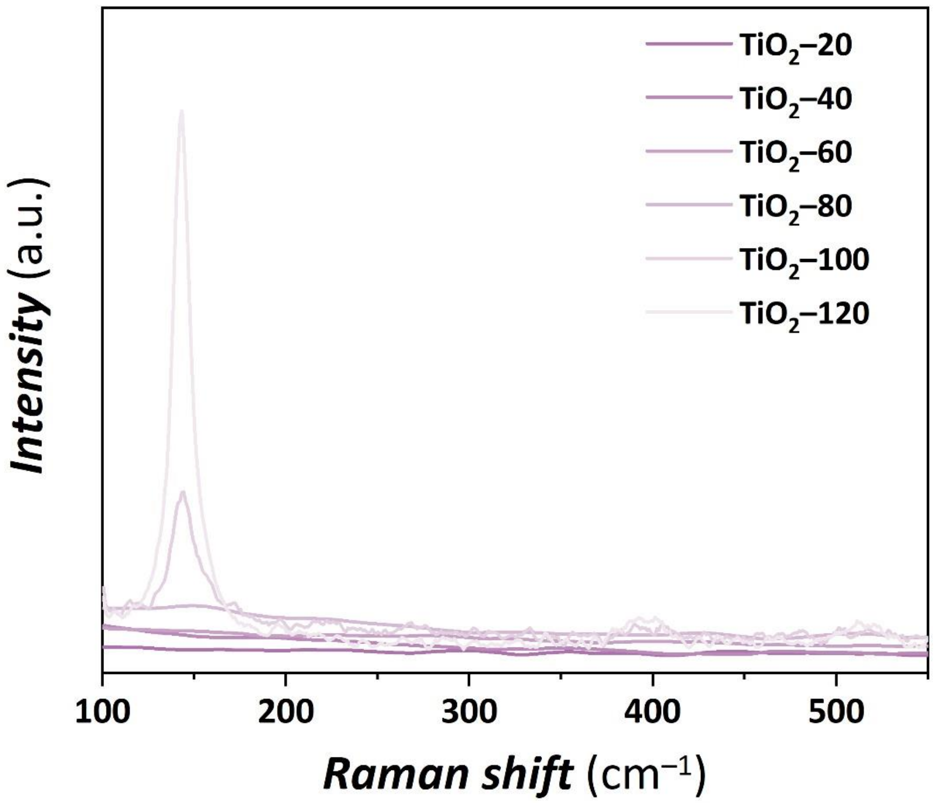

Figure S9. Raman spectra of anodic TiO_2 nanofilms, from TiO_2–20 to TiO_2–120.

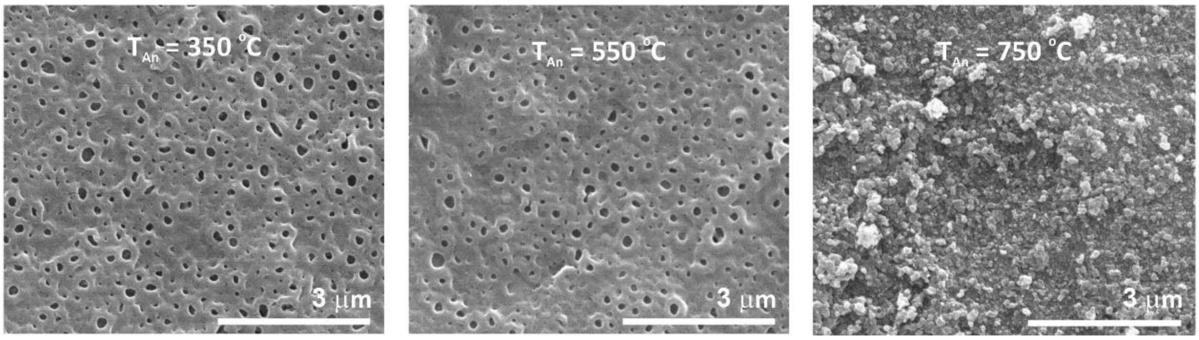

Figure S10. Top view FEG-SEM images of TiO_2–120 nanofilms at distinct annealing temperatures, from 350 to 750 ºC.

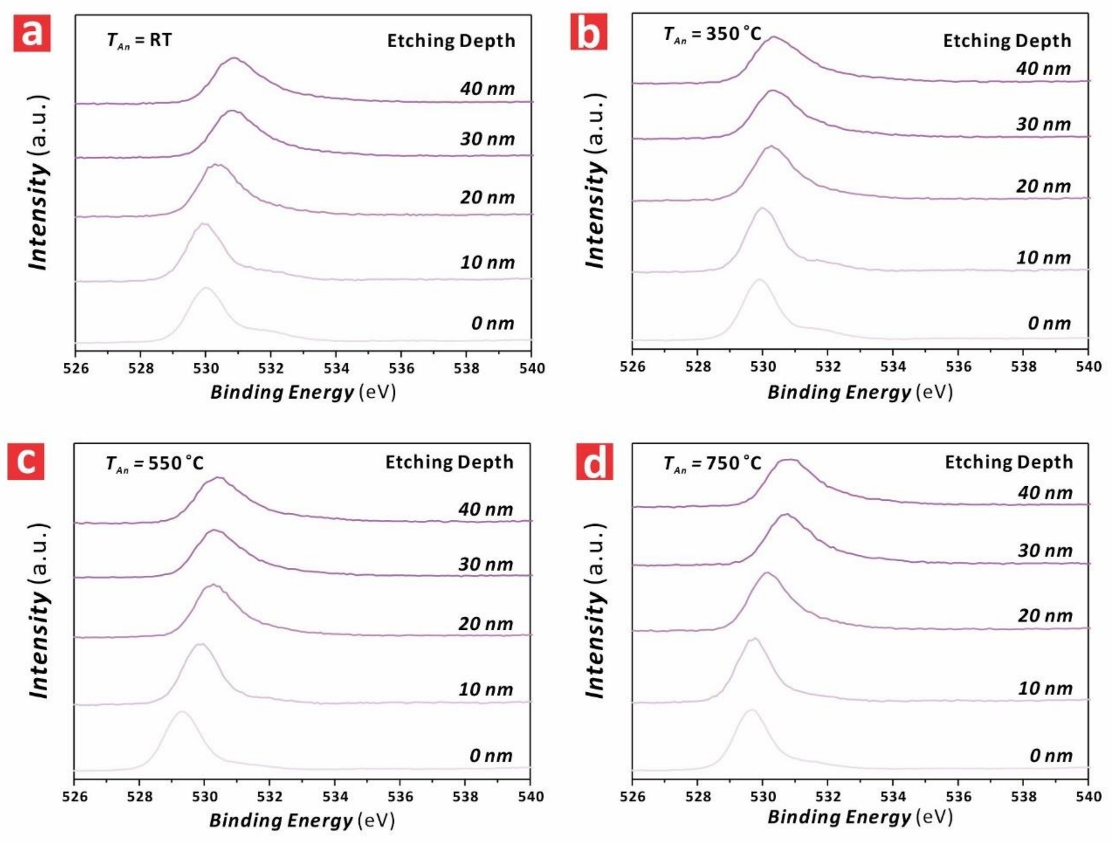

Figure S11. O 1s XPS spectra of TiO_2–120 nanofilms annealed at T_{An} = (a) RT, (b) 350, (c) 550 and (d) 750 °C, analyzed at distinct nanofilm thickness depth, from 0 to 40 nm at steps of 10 nm.

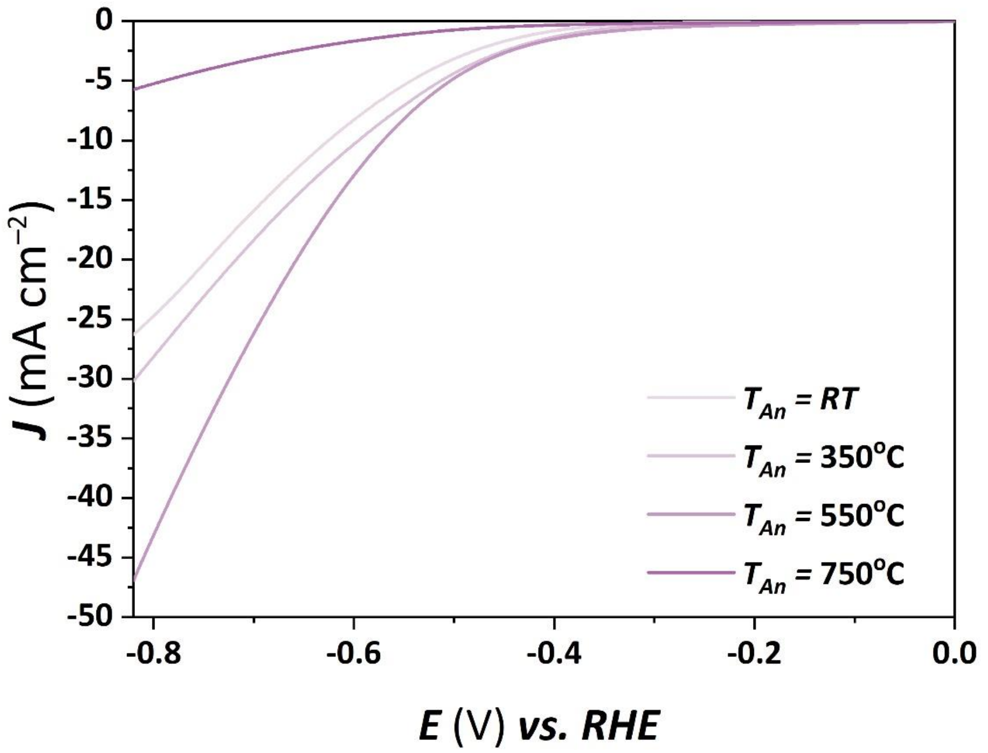

Figure S12. Linear sweep voltammograms of anodic TiO_2–120 nanofilms under non-illumination conditions annealed at T_{An} = RT, 350, 550 and 750 °C under varying overpotential (*E*), from –0.82 to 0.0 V *vs* RHE, at a rate of 0.005 V s^{-1} in 1 M KOH.

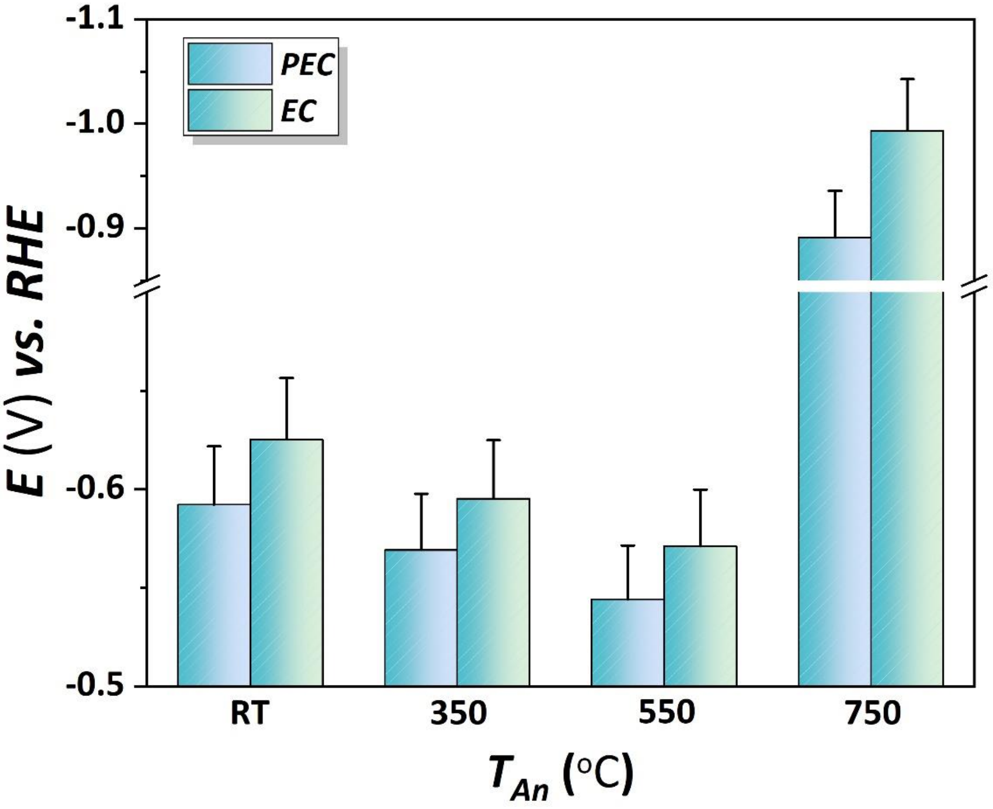

Figure S13. Overpotential values *vs* RHE measured in anodic TiO_2–120 nanofilms annealed at T_{An} = RT, 350, 550 and 750 °C under illumination and non-illumination conditions to deliver a current density of J = 10 mA cm^{-2} in 1 M KOH (i.e., performance metric for HER) (NB: data are presented as mean ± SD of a sample size of $n \geq 3$ independent measurements).

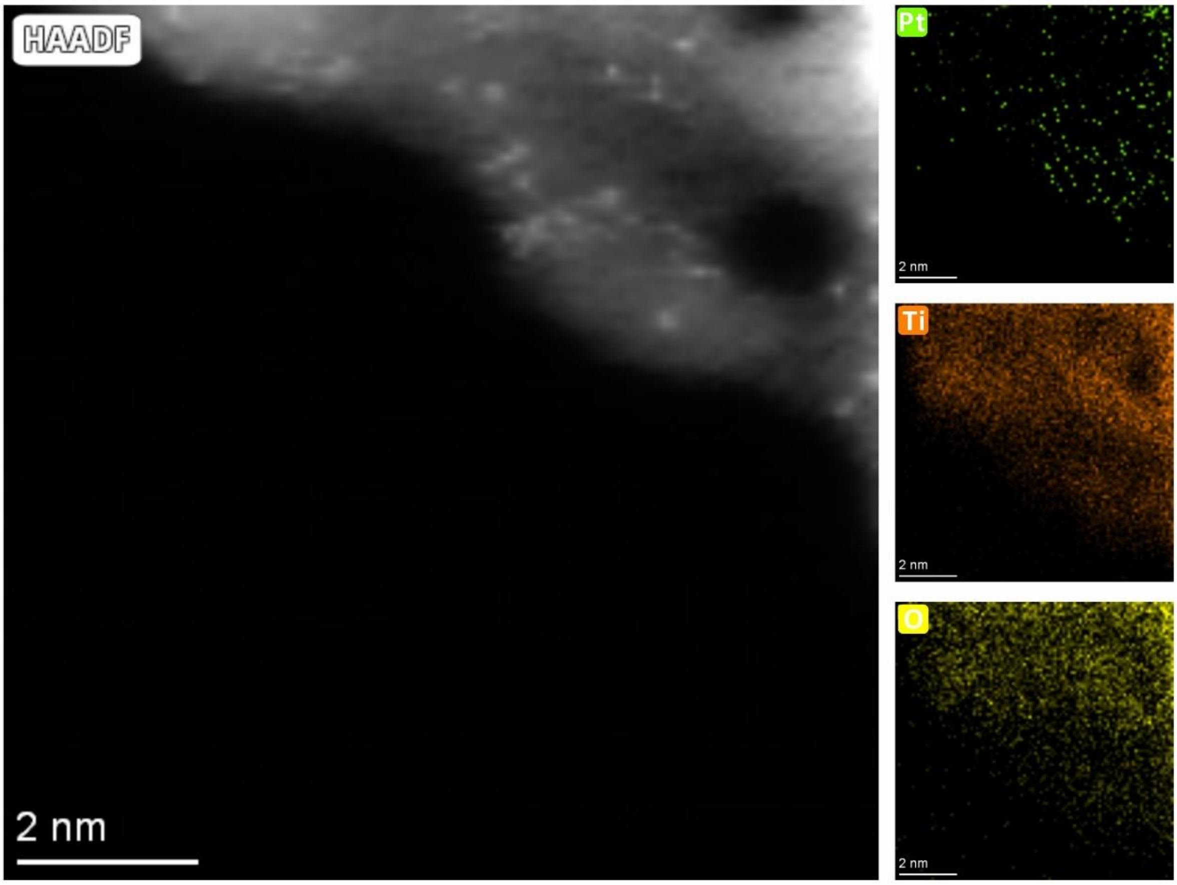

Figure S14. HAADF-TEM images of an anodic TiO_2–120 nanofilm annealed at 550 ^{o}C and modified with SAC at *[Pt]* = 0.01 mM (scale bar = 2 nm).

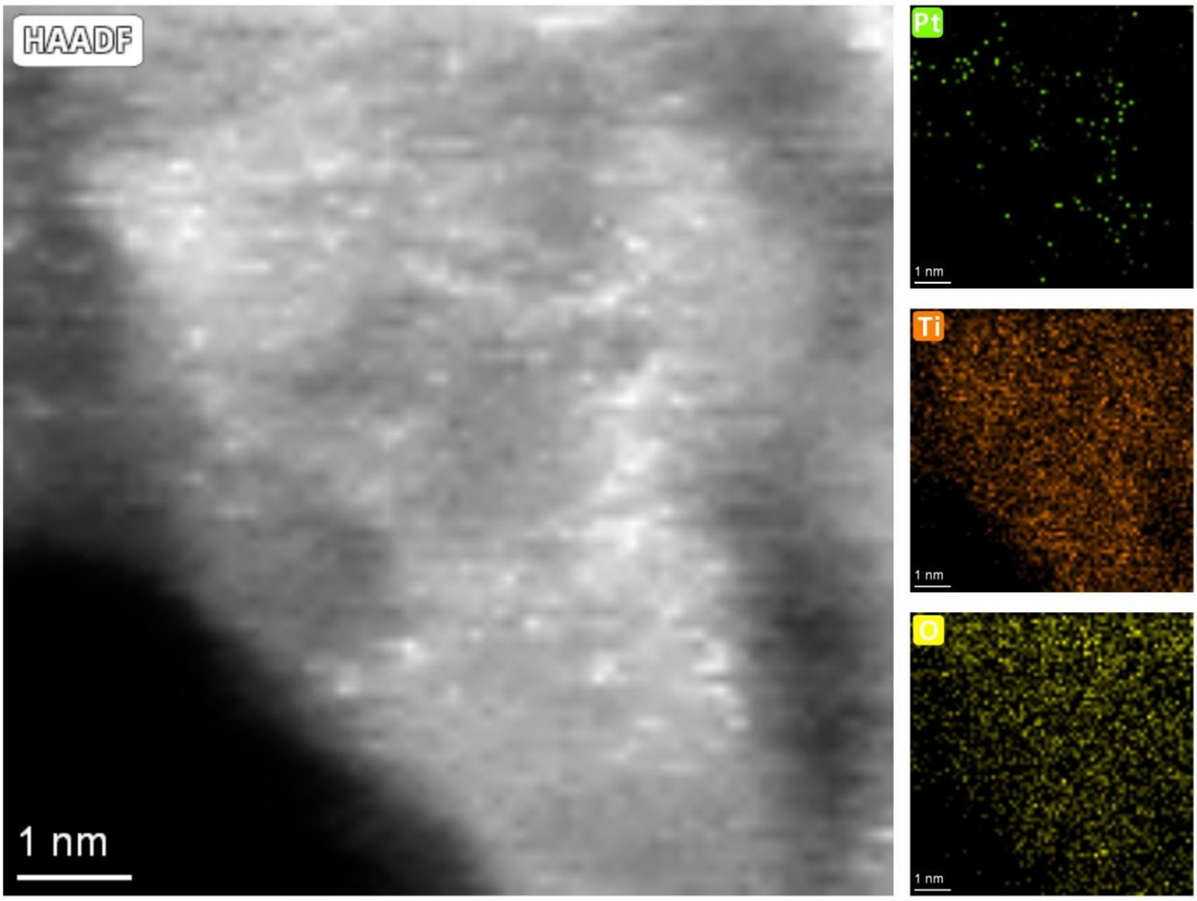

Figure S15. HAADF-TEM images of an anodic TiO_2–120 nanofilm annealed at 550 ^oC and modified with SAC at *[Pt]* = 0.01 mM (scale bar = 1 nm).

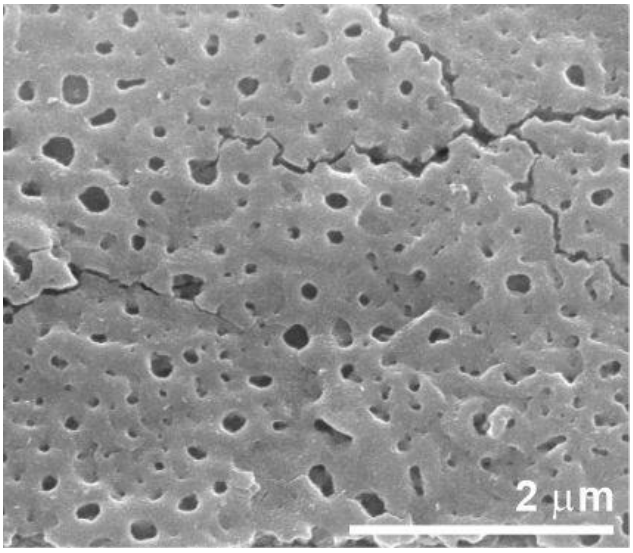

Figure S16. Top-view FEG-SEM images of an anodic TiO_2–120 nanofilm annealed at 550 ºC and modified with SAC at *[Pt]* = 0.01 mM after 5000 HER cycle stability test.

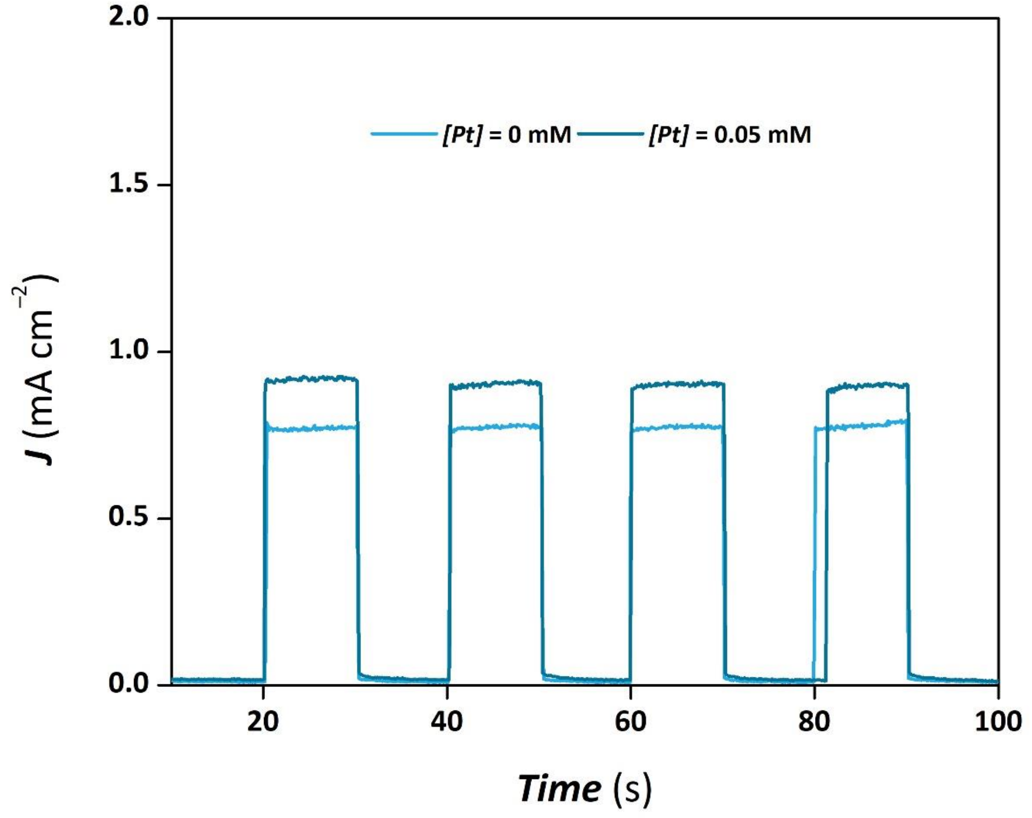

Figure S17. Chronoamperometry of an anodic TiO_2–120 nanofilm annealed at 550 °C and modified with SAC at *[Pt]* = 0 (as-produced) and 0.05 mM.

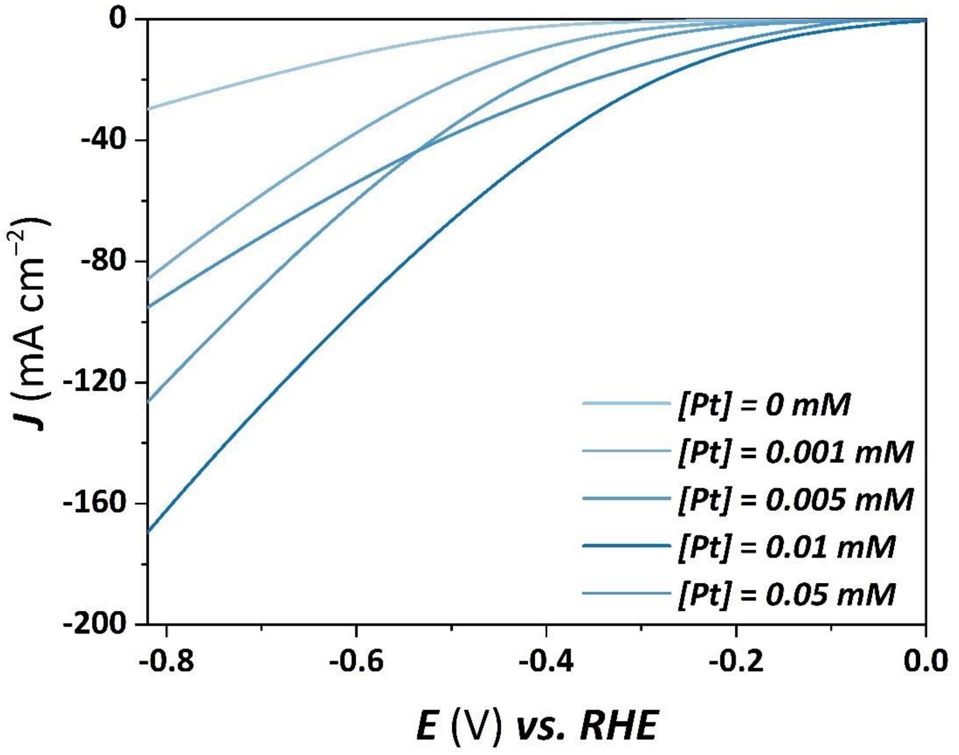

Figure S18. Linear sweep voltammograms of TiO_2–120 nanofilms annealed at 550 °C under non-illuminated conditions and modified with single-atom Pt catalyst atoms at varying concentration (i.e., *[Pt]* = 0, 0.001, 0.005, 0.01 and 0.05 M) under varying overpotential (*E*), from –0.82 to 0.0 V *vs* RHE, at a rate of 0.005 V s^{-1} in 1 M KOH.

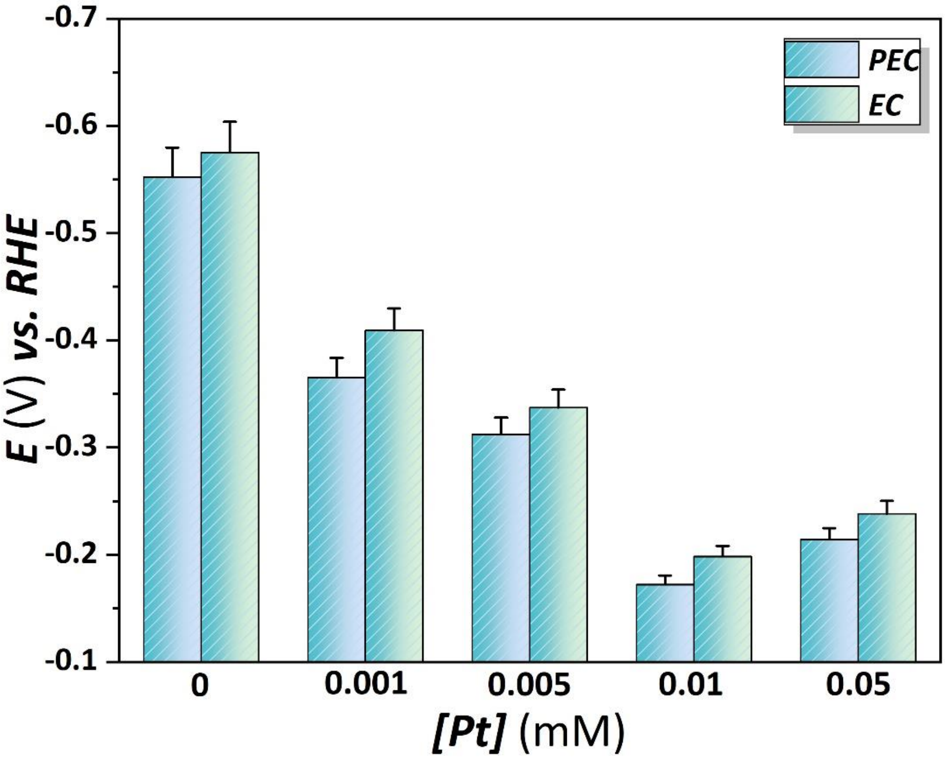

Figure S19. Overpotential values *vs* RHE measured TiO_2–120 nanofilms annealed at 550 °C under illumination and non-illumination conditions modified with single-atom Pt catalyst atoms at varying concentration (i.e., *[Pt]* = 0, 0.001, 0.005, 0.01 and 0.05 M) to deliver a current density of J = 10 mA cm^{-2} in 1 M KOH (i.e., performance metric for HER) (NB: data are presented as mean ± SD of a sample size of $n \geq 3$ independent measurements).

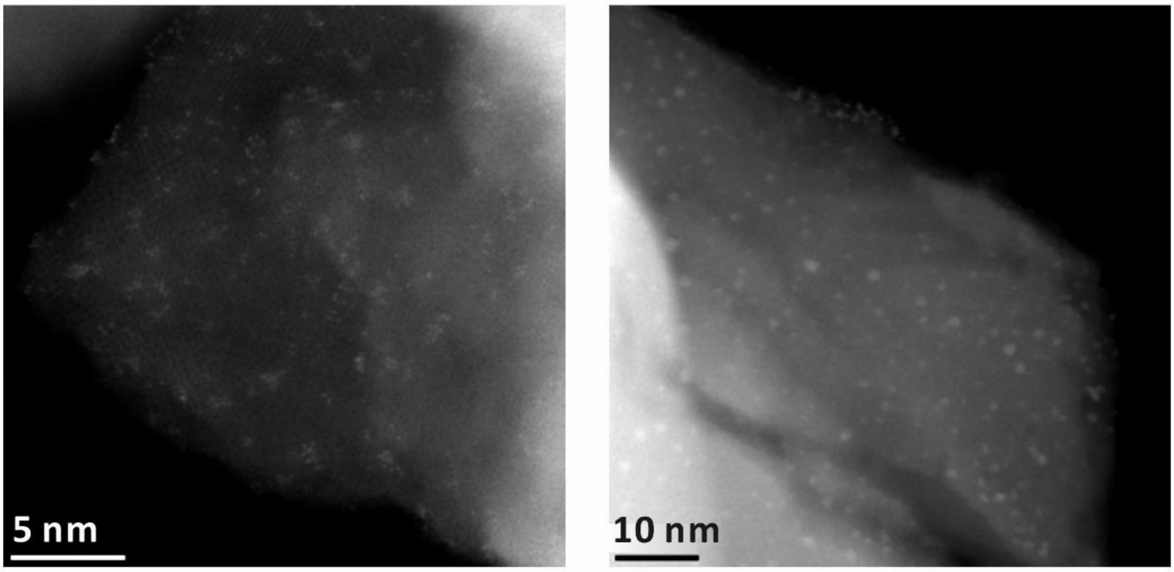

Figure S20. HAADF-TEM images of a representative anodic TiO_2–120 nanofilms annealed at 550 °C modified with single-atom Pt catalyst via dark deposition using a precursor with a concentration of *[Pt]* = 0.05 mM (NB: Left figure contains single atom Pt and right figure contains more Pt clusters).

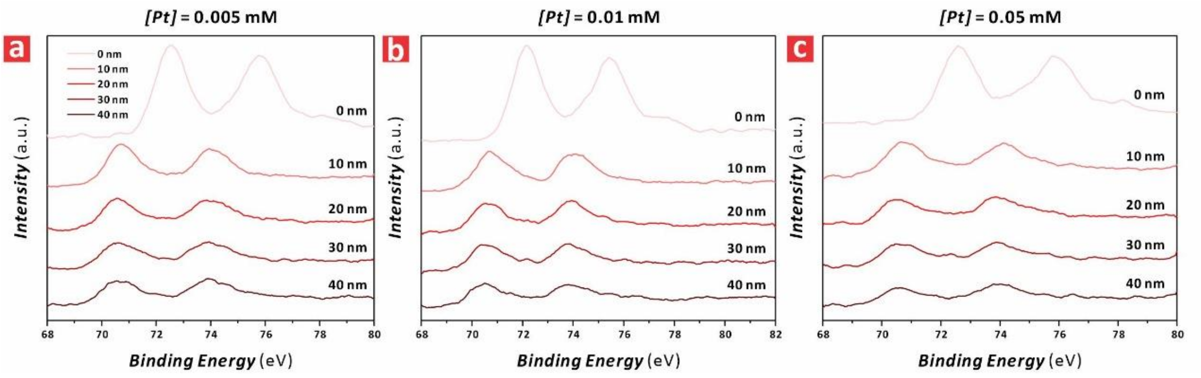

Figure S21. XPS spectra of Pt 4f of etched TiO_2–120 nanofilms annealed at T_{An} 550 °C within etching depth of 10, 20, 30 and 40 nm and modified with single-atom Pt catalyst via dark deposition using a precursor with a concentration of: (a) *[Pt]* = 0.005 mM; (b) *[Pt]* = 0.01 mM; and (c) *[Pt]* = 0.05 mM (NB: etching rate = 0.25 nm s^{-1}).

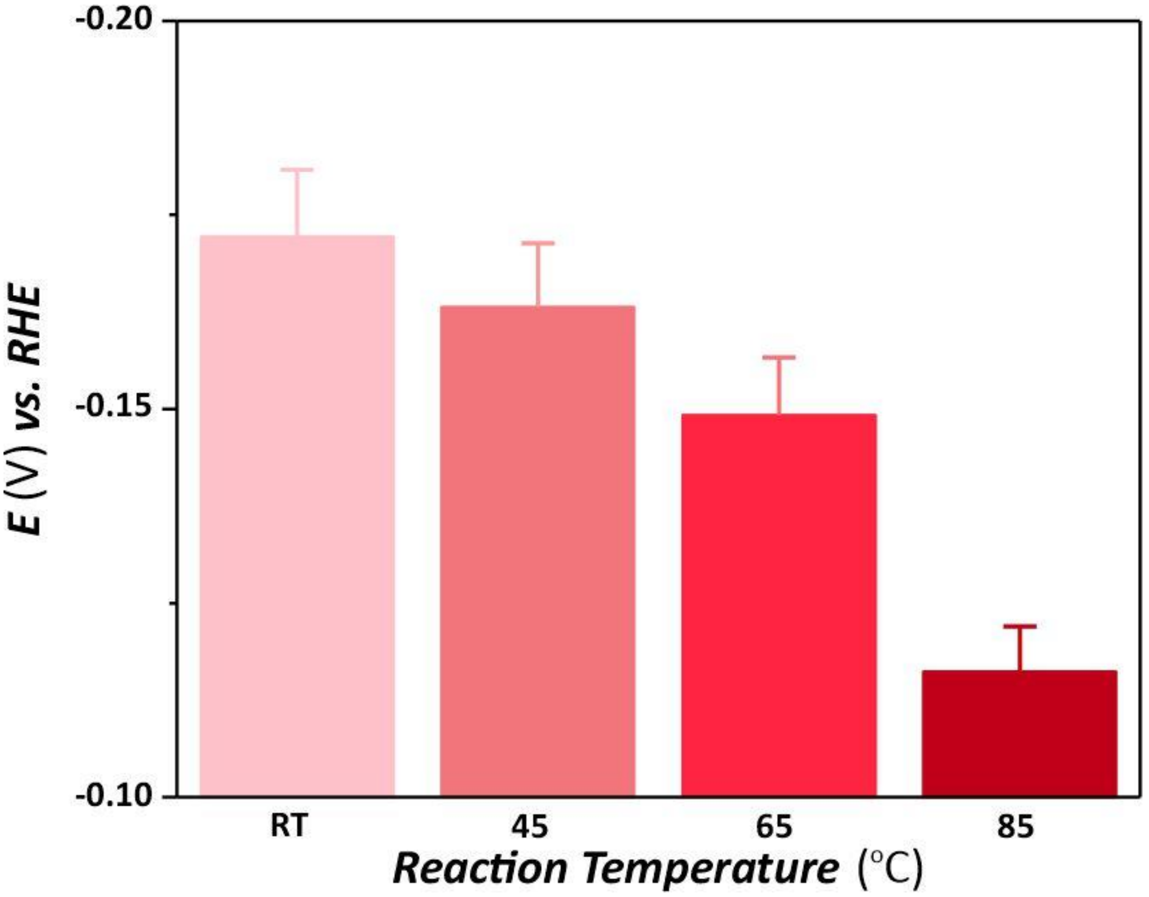

Figure S22. Overpotential values *vs* RHE measured in TiO_2–120 nanofilms annealed at T_{An} 550 °C and modified with single-atom Pt catalyst at varying reaction temperature (T = 25, 45, 65 and 85 °C) to deliver a current density of J = 10 mA cm^{-2} in 1 M KOH (i.e., performance metric for HER) (NB: data are presented as mean ± SD of a sample size of $n \geq 3$ independent measurements).

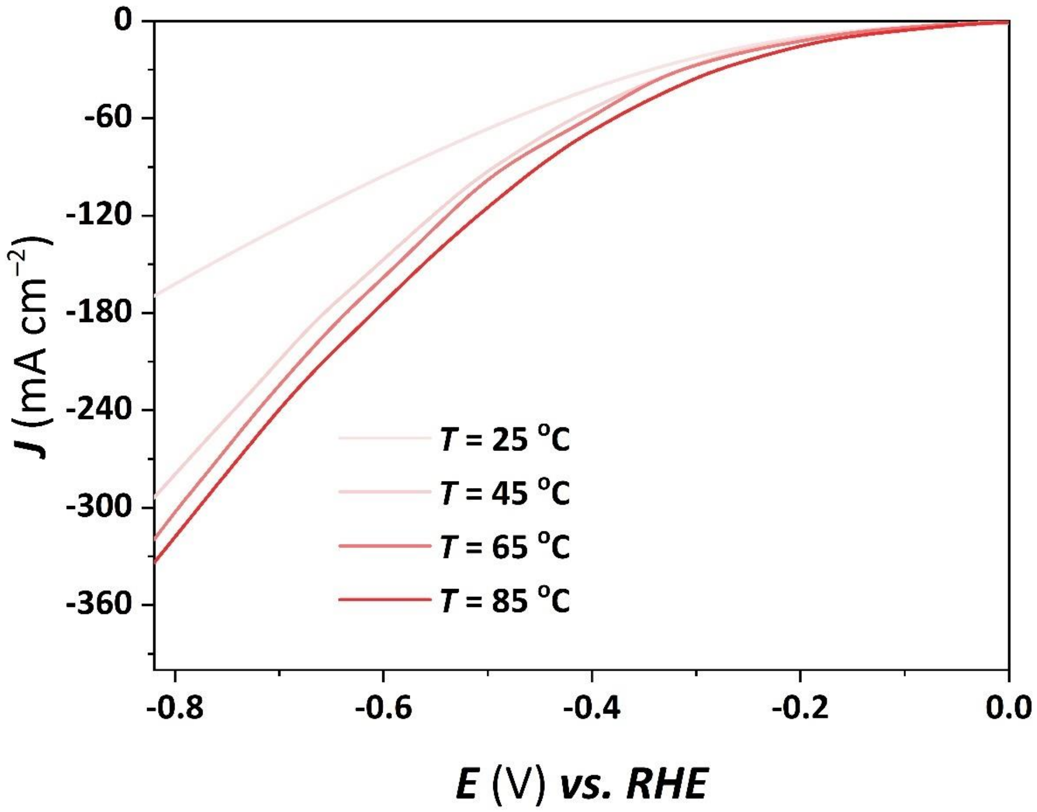

Figure S23. Linear sweep voltammograms of TiO_2–120 nanofilms annealed at T_{An} 550 °C and modified with single-atom Pt catalyst atoms at varying reaction temperature (*T* = 25, 45, 65 and 85 °C) under varying overpotential (*E*), from –0.82 to 0.0 V *vs* RHE, at a rate of 0.005 V s^{-1} in 1 M KOH.

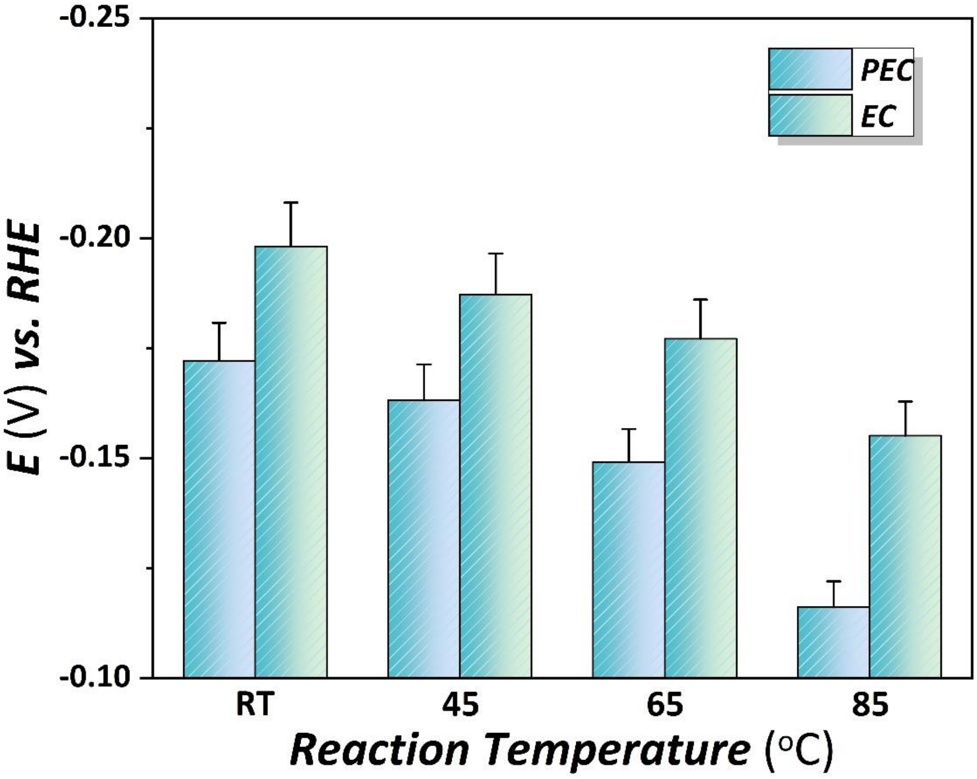

Figure S24. Overpotential values *vs* RHE measured in TiO_2–120 nanofilms annealed at T_{An} 550 °C and modified with single-atom Pt catalyst atoms under illuminated and non-illuminated at varying reaction temperature (T = 25, 45, 65 and 85 °C) to deliver a current density of J = 10 mA cm^{-2} in 1 M KOH (i.e., performance metric for HER) (NB: data are presented as mean ± SD of a sample size of $n \geq 3$ independent measurements).

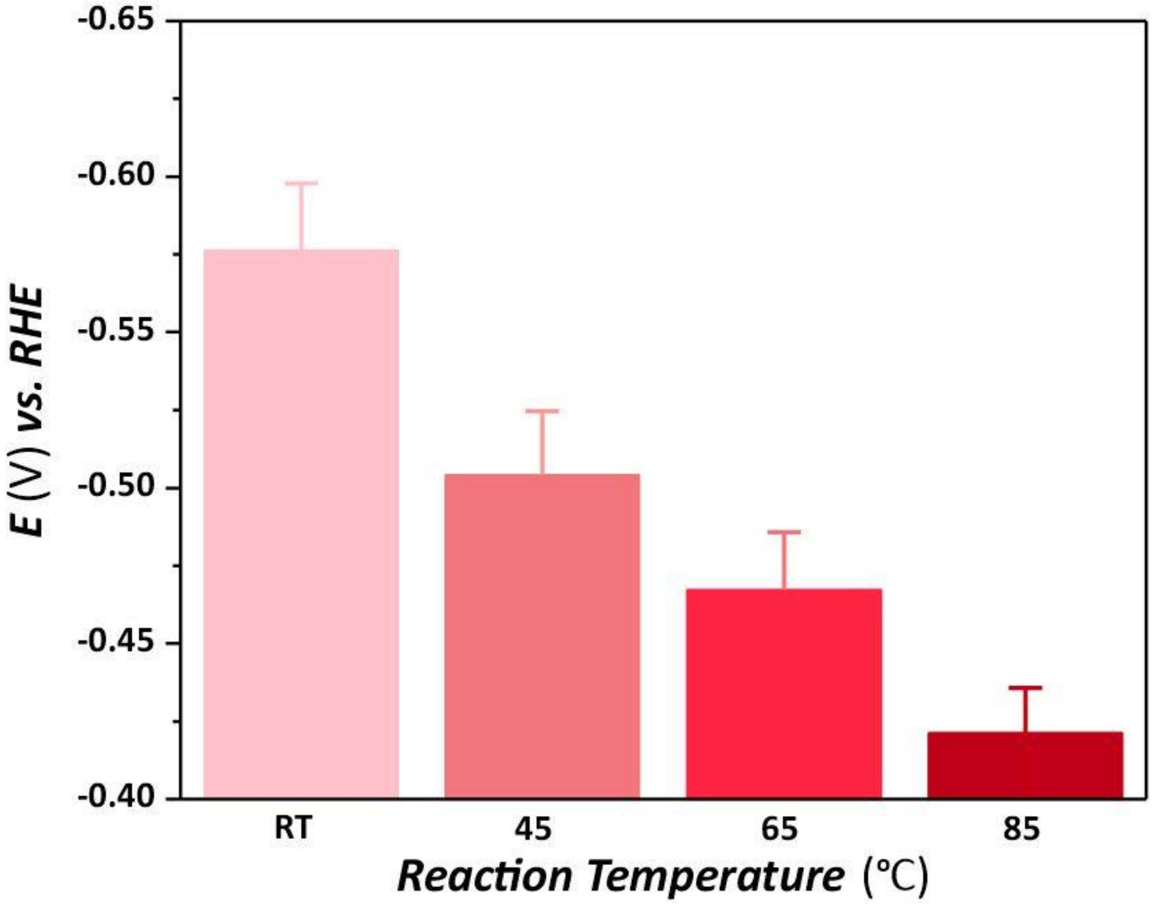

Figure S25. Overpotential values *vs* RHE measured in TiO_2–120 nanofilms annealed at T_{An} 550 °C and modified with single-atom Pt catalyst atoms at varying reaction temperature (T = 25, 45, 65 and 85 °C) to deliver a current density of J = 100 mA cm^{-2} in 1 M KOH (i.e., performance metric for HER) (NB: data are presented as mean ± SD of a sample size of $n \geq 3$ independent measurements).

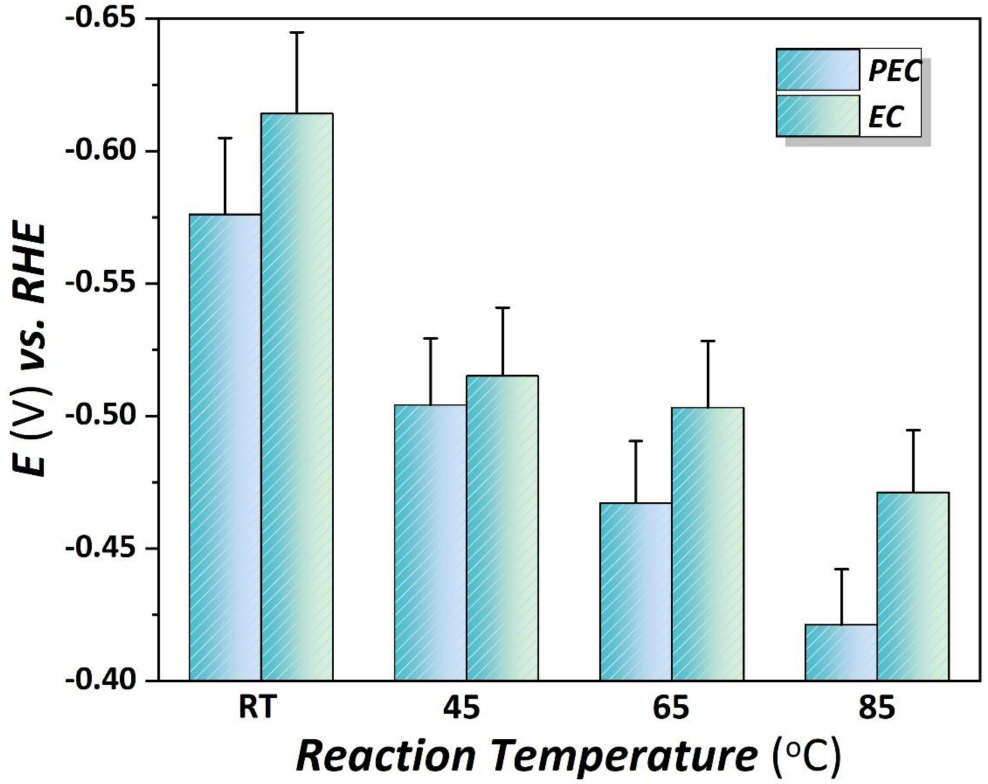

Figure S26. Overpotential values *vs* RHE measured in TiO_2–120 nanofilms annealed at T_{An} 550 °C and modified with single-atom Pt catalyst atoms under illuminated and non-illuminated at varying reaction temperature (T = 25, 45, 65 and 85 °C) to deliver a current density of J = 100 mA cm^{-2} in 1 M KOH (i.e., performance metric for HER) (NB: data are presented as mean ± SD of a sample size of $n \geq 3$ independent measurements).

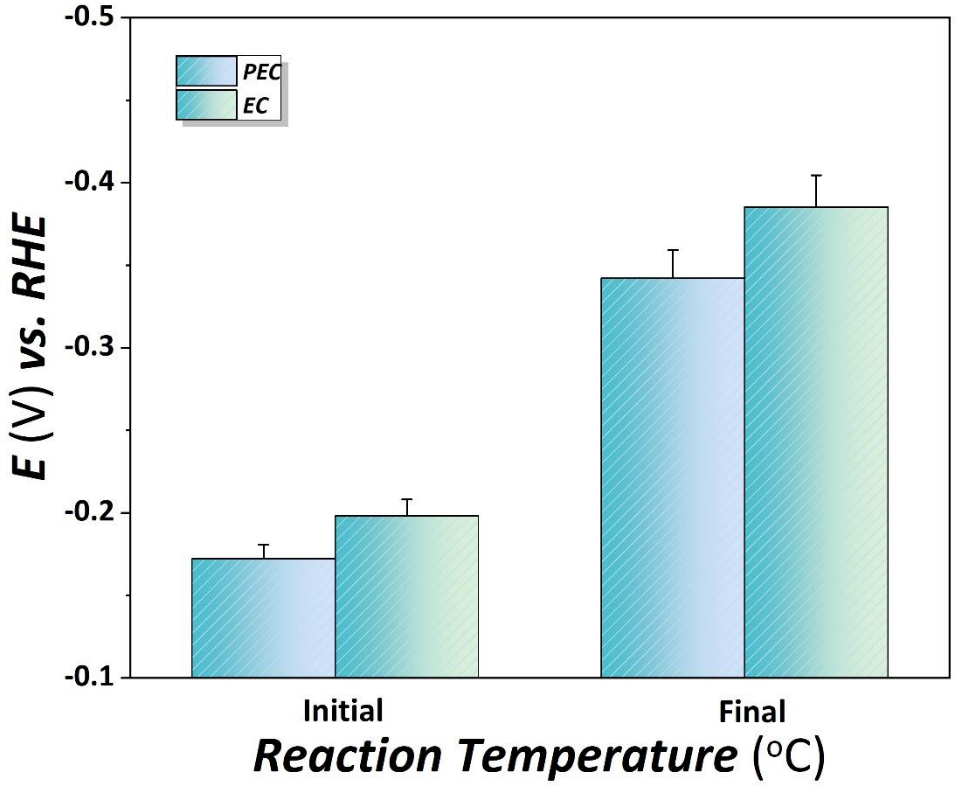

Figure S27. Overpotential values *vs* RHE measured in TiO_2–120 nanofilms annealed at T_{An} 550 °C and modified with single-atom Pt catalyst atoms under illuminated and non-illuminated before and after thermal reaction to deliver a current density of $J = 10$ mA cm^{-2} in 1 M KOH (i.e., performance metric for HER) (NB: data are presented as mean ± SD of a sample size of $n \geq 3$ independent measurements).

Table S1. Values of electronic components of equivalent circuit of EIS for: TiO_2–20 at V_{an} = 20 V; TiO_2–40 at V_{an} = 40 V; TiO_2–60 at V_{an} = 60 V; TiO_2–80 at V_{an} = 80 V; TiO_2–100 at V_{an} = 100 V; and TiO_2–120 at V_{an} = 120 V.

V_{an} (V)	20	40	60	80	100	120
R_1 (Ω)	16.3	17.2	20	23.92	13.56	19.49
Q_2 ($\mu F \cdot s^{a-1}$)	516.4	1144	2392	2445	2159	2009
a_2	0.62	0.61	0.56	0.67	0.72	0.75
R_2 (Ω)	4377	47976	3874	2999	19561	4651
Q_3 ($\mu F \cdot s^{a-1}$)	15.09	21.79	57.41	135	178.7	103.4
a_3	0.96	0.94	0.92	0.84	0.8031	0.8697
R_3 (Ω)	174.5	130.6	69.35	64.35	97.61	79.89

Table S2. ICP–MS of TiO_2–120 nanofilms modified with single-atom Pt catalyst atoms at varying concentration of *[Pt]* = 0.005, 0.01 and 0.05 mM.

**[Pt]** (mM)	m_0 (g)	V_0 (mL)	C_0 (mg L^{-1})	**f**	C_1† (mg L^{-1})	C_x‡ (mg kg^{-1})	**W** (%)[1]
0.05	0.20	10	0.13	1	0.13	6.24	0.0006
0.01	0.19	10	0.02	1	0.02	1.03	0.0001
0.005	0.19	10	0.05	1	0.05	2.81	0.0003

Table S3. ICP–MS of TiO_2 nanofilms modified with single-atom Pt catalyst atoms before and after thermal reaction.

**States**	m_0 (g)	V_0 (mL)	C_0 (mg L^{-1})	**f**	C_1† (mg L^{-1})	C_x‡ (mg kg^{-1})	**W** (%)[1]
Initial	0.20	10	0.13	1	0.13	6.24	0.0006
Final	0.21	10	0.04	1	0.04	1.93	0.0002

† C_1 is calculated using the following equation $C_1\left(\frac{mg}{L}\right) = C_0\left(\frac{mg}{L}\right) * f$

‡ C_x is calculated based on $Cx\left(\frac{mg}{kg}\right) = \frac{C_0\left(\frac{mg}{L}\right)*f*V_0(mL)*10^{-3}}{m(g)*10^{-3}} = \frac{C_1\left(\frac{mg}{L}\right)*V_0(mL)*10^{-3}}{m(g)*10^{-3}}$

[1] W is determined by $W(\%) = \frac{Cx\left(\frac{mg}{kg}\right)}{10^6} * 100\%$

Where m_0 is the weight of samples, V_0 is the volume of dissolved sample solution, $C_0$is the tested concentration of solution elements, f is dilution factor, $C_1$is elemental concentration of sample digestion solution, C_x is final test results for the measured elements and W is the mass fraction of measured elements.

Table S4. Gibbs free energy of hydrogen adsorption of anatase TiO_2 via increased reaction temperature from room temperature to 85 °C.

**Temperature** (°C)	**Gibbs free energy (G_{ad})**			
	RT	45	65	85
Anatase	-0.32364	0.101559	0.171741	-0.47879
Anatase + SA Pt	-0.31074	0.113359	0.184725	-0.465355

Table S5. The structure parameters of Anatase (001).

	Anatase (001)
Cell length (a.b.c)(Å)	a = b = 7.734, c = 39.390
Cell angle (°)	α = β = γ = 90.00
Vacuum space (Å)	20
TiO2 layers (frozen/relaxed)	4/4

References

[1] S. Franz, H. Arab, G. L. Chiarello, M. Bestetti, E. Selli, *Adv. Energy Mater.* **2020**, 10, 2000652.
